# Syntheses, Structural Characterization, and Cytotoxicity Assessment of Novel Mn(II) and Zn(II) Complexes of Aroyl-Hydrazone Schiff Base Ligand

**DOI:** 10.1021/acsomega.2c05927

**Published:** 2023-01-11

**Authors:** Masrat Bashir, Aijaz A. Dar, Imtiyaz Yousuf

**Affiliations:** † Department of Chemistry, Aligarh Muslim University, Aligarh202002, Uttar Pradesh, India; ‡ Department of Chemistry, University of Kashmir, Hazratbal, Srinagar190006, Jammu & Kashmir, India

## Abstract

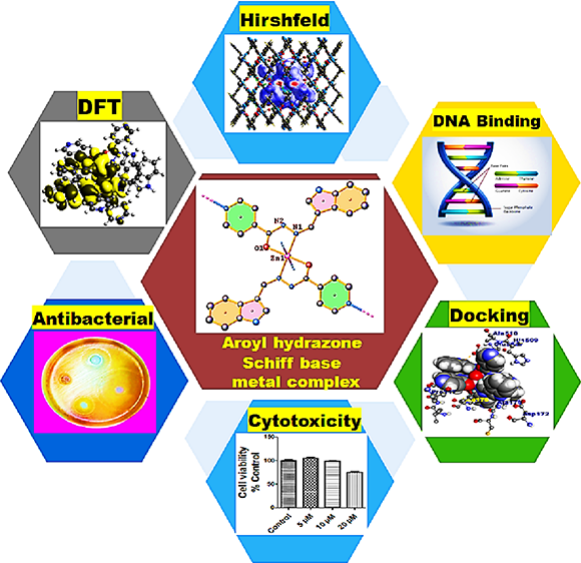

This work describes the syntheses, structural characterization, and biological profile of Mn(II)- and Zn(II)-based complexes **1** and **2** derived from the aroyl-hydrazone Schiff base ligand (**L1**). The synthesized compounds were thoroughly characterized by elemental analysis, Fourier transform infrared spectroscopy (FTIR), UV–vis, electron paramagnetic resonance (EPR), nuclear magnetic resonance (NMR), and single-crystal X-ray diffraction (s-XRD). Density functional theory (DFT) studies of complexes **1** and **2** were performed to ascertain the structural and electronic properties. Hirshfeld surface analysis was used to investigate different intermolecular interactions that define the stability of crystal lattice structures. To ascertain the therapeutic potential of complexes **1** and **2**, *in vitro* interaction studies were carried out with ct-DNA and bovine serum albumin (BSA) using analytical and multispectroscopic techniques, and the results showed more avid binding of complex **2** than complex **1** and **L1**. The antioxidant potential of complexes **1** and **2** was examined against the 2,2-diphenyl picrylhydrazyl (DPPH) free radical, which revealed better antioxidant ability of the Mn(II) complex. Moreover, the antibacterial activity of synthesized complexes **1** and **2** was tested against Gram-positive and Gram-negative bacteria in which complex **2** demonstrated more effective bactericidal activity than **L1** and complex **1** toward Gram-positive bacteria. Furthermore, the *in vitro* cytotoxicity assessment of **L1** and complexes **1** and **2** was carried out against MDA-MB-231 (triple negative breast cancer) and A549 (lung) cancer cell lines. The cytotoxic results revealed that the polymeric Zn(II) complex exhibited better and selective cytotoxicity against the A549 cancer cell line as was evidenced by its low IC_50_ value.

## 1 Introduction

The successful clinical translation of platinum complexes, viz., cisplatin, oxaliplatin, and carboplatin, as prospective anticancer drugs has resulted in a significant dominance of platinum drugs in the chemotherapeutic drug regime.^1^ Although cisplatin has been phenomenal against solid malignancies including bladder, ovarian, and testicular cancers, the severe side effects, intrinsic drug resistance, and general toxicity have resulted in its limited use.^2^ As a result, the past few decades have witnessed an extensive exploration of non-platinum complexes especially based on 3d-metal ions as potential therapeutic agents.^3,4^ The first-row transition-metal elements have received special consideration on account of their diverse features, which include physiologically tunable oxidation states, rich redox chemistry, lesser toxic behavior, and target specific interactions with the biomolecules.^5^ Besides the variability in the oxidation state and redox properties, the diverse molecular architecture exhibited by first-row transition metals owing to their wide range of geometries and coordination numbers often modulates their kinetic (rates of ligand exchange) and thermodynamic reactivity with the targeted biomolecules.^6^ The redox active metals of first-row transition elements, especially Mn, Fe, Co, and Cu, comprise an integral part of proteins and enzymes, thereby offering electron transfer as well as catalytic and structural roles.^7^

Manganese is a bioessential trace element and offers a wide range of stable oxidation states (0 to VII); nevertheless, in biological systems, it is mostly present in II, III, and IV oxidation states.^8^ Manganese is essentially present in the active sites of specific enzymes like catalase (Mn-CAT) and superoxide dismutase (Mn-SOD), thereby regulating the scavenging of reactive oxygen species (ROS) in oxidative stress.^9^ The catalase and superoxide dismutase enzymes exert their antioxidant catalytic functions in a way of disproportionation of detrimental H_2_O_2_ (into water and dioxygen) and dismutation of superoxide ions, respectively.^10^ Notably, many literature reports have revealed transferrin receptor (TfR) proteins (which are highly expressed in tumor cells) essential for the cellular uptake and transport of Mn(II) ions *in vivo*.^11^ Therefore, it can be speculated that Mn(II) complexes could exert their antitumor action by specifically impairing the transport mechanism of TfR proteins.^12^ In recent years, various binuclear Mn(II) complexes have been synthesized with diverse ligand scaffolds and shown to exhibit remarkable cytotoxic activity *in vitro*.^13,14^

Zinc features as the 2nd most prevalent trace-metal element in the human body and is actively involved in the DNA synthesis and repair mechanisms, cell metabolism, and defense against oxidative damage.^4^ Zn(II) serves as the main component of a large number of enzymes offering structural, catalytic, and cocatalytic functions due to its unique physicochemical properties, Lewis acid character, and ability to stabilize various coordination geometries amenable to ligand exchange.^15^ Many Zn(II) complexes have been exploited for their potential pharmacological properties, viz., anticonvulsant, antidiabetic, anti-inflammatory, and antimicrobial, besides being used in the treatment of neurodegenerative diseases.^16,17^ Recent studies have shown that Zn(II) derivatives have proven to be effective anticancer agents that typically exhibit lower *in vivo* side effects apart from eliciting different modes of anticancer action in contrast to classical metal-based chemotherapeutic drugs.^18^

The choice of an appropriate ligand framework in a metal complex plays a decisive role in modulating pharmacological properties by altering the reactivity or substitution inertness, balancing lipid/water solubility, limiting the side effects of metal-ion overload, and facilitating metal-ion distribution *in vivo*.

Schiff bases feature as a special class of organic ligands on account of their flexible entanglement, structural divergence, and facile coordination to metal ions. Schiff base ligands have gained significant importance because of azomethine (−C=N−) linkages, which offer a supportive role in stability, chelating ability, and favorable biological properties. A rationally designed Schiff base ligand scaffold can improve the therapeutic profile of metal complexes by favorably modulating the hard/soft property of coordinating metal ions and the lipophilic/hydrophilic balance of the resulting complex.^19^ Hydrazone-based Schiff base ligands, especially aroyl-hydrazones, which contain a basic unit of ArCH=NNHC(=O)Ar′ (Ar = aromatic ring), belong to a special class of chelating (bi- or tridentate) azomethine ligands.^20^ Apart from displaying versatility and flexibility in their structures, aroyl-hydrazone compounds are known for their efficient pharmacological properties including anticonvulsant, antidepressant, analgesic, antimicrobial, antiviral, and antitumor behaviors.^21,22^

Thus, it is imperative to modulate the pharmacological properties of aroyl-hydrazone ligands by incorporating a lesser toxic metal ion that could possibly amplify the therapeutic and targeted properties of the prospective metal complex. Herein, in this work, we report the synthesis of two novel Mn(II)/Zn(II) complexes derived from the aroyl-hydrazone Schiff base ligand (**L1**) as efficient and less toxic chemotherapeutic agents. To validate the chemotherapeutic potential of ligand **L1** and Mn(II) and Zn(II) complexes, we carried out DNA/BSA binding studies and examined their cytotoxicity against A549 and MDA-MB-231 cancer cells.

## 2 Results and Discussions

### 2.1 Synthesis and Characterization

**Scheme 1:**
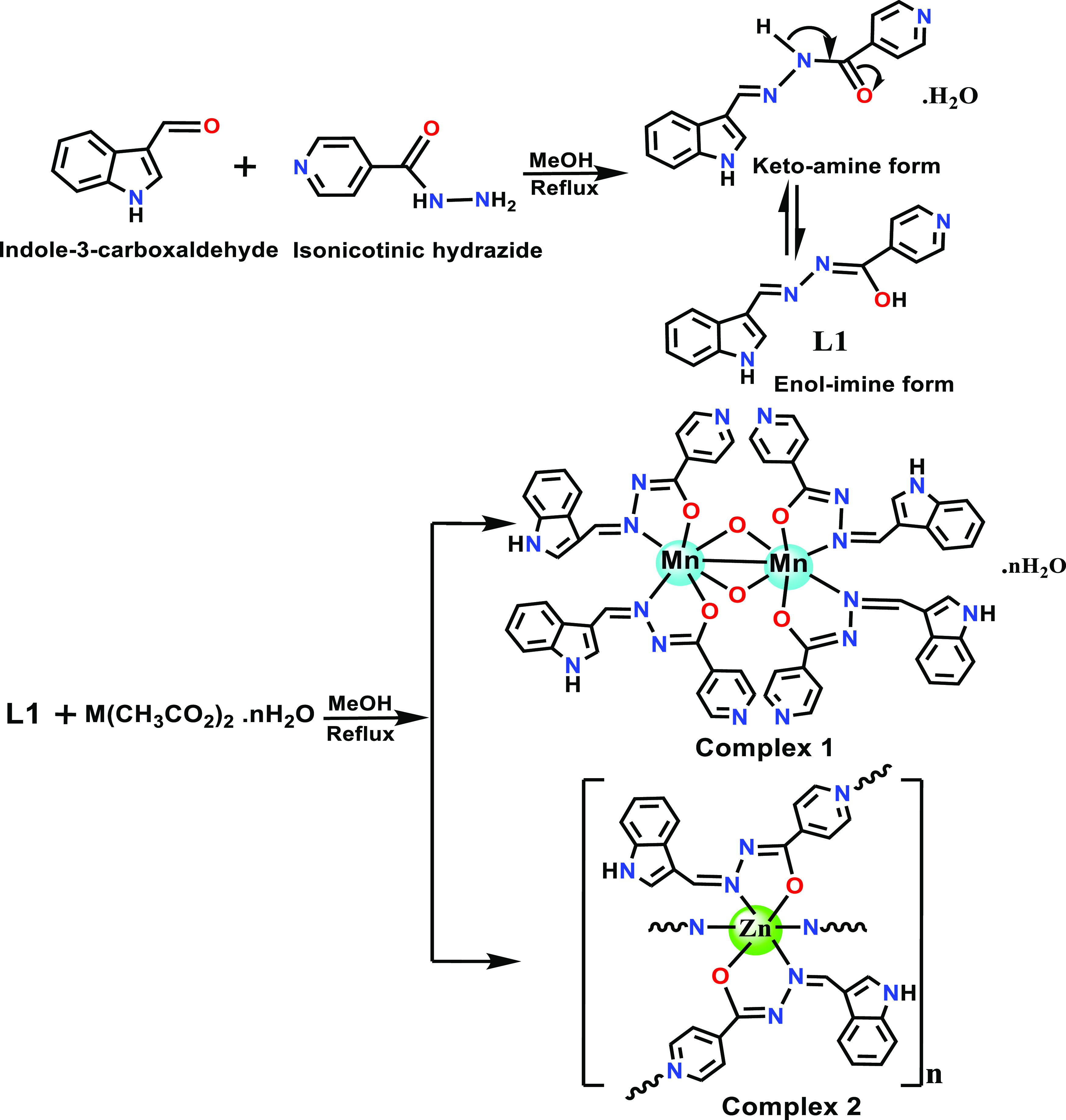
Synthetic Route for the Formation of **L1** and Complexes **1** and **2**

The aroyl-hydrazone Schiff base ligand (**L1**) was synthesized by earlier reported methods by refluxing the methanolic solutions of indole-3-carboxaldehyde and isonicotinic hydrazide in a 1:1 stoichiometric ratio.^23^ The complexation of **L1** was carried out by adding methanolic solutions of respective Mn(II) (complex **1**) and Zn(II) (complex **2**) acetate as a metal precursor salt in a 2:1 stoichiometric ratio, as described in Scheme 1. The molecular structure of **L1** and the corresponding metal complexes **1** and **2** were validated by analytical and spectroscopic methods, viz., FTIR, EPR, UV–vis, and NMR, which are supported well by single-crystal X-ray structures. Moreover, complexes **1** and **2** were found to be fairly soluble in CH_3_OH, DMSO, and DMF solvents and partially soluble in water. The solution stability studies of complexes **1** and **2** were ascertained against varied time intervals (0–72 h) under physiological conditions by employing electronic absorption spectroscopy (Figure S1). Notably, even after a period of 72 h, no discernible shift in the intensity or position of absorbance bands was observed, thereby validating the solution stability of complexes **1** and **2** for a substantial period of time.

Electronic spectrum of the ligand **L1** revealed intraligand transition bands at 265 and 327 nm, which are ascribed to π–π* and n−π* transitions of aromatic chromophore and azomethine groups, respectively, and were subsequently shifted upon coordination with the metal ions (Figure S2). Moreover, no d–d band was observed for complexes **1** and **2** because Mn(II) is in the d^5^ electronic configuration with ^6^A_1g_–^4^T_1g_ spin-forbidden transition, while the Zn(II) ion has a complete d^10^ electronic configuration.^24,25^ The effective magnetic moment value of complex **1** was evaluated using the equation μ_eff_ = 2.82[χ_m_T]^1/2^, and the value was found to be 5.49 B.M, validating the high spin nature of complex **1** with five unpaired electrons.^26^

### 2.2 FTIR Spectroscopy

The comparative FTIR spectral data of **L1** and complexes **1** and **2** were used to validate the coordination mode of the free ligand with the metal ions. In the FTIR spectrum of free ligand **L1**, the characteristic IR bands exhibited at 3396, 1657, and 1602 cm^–1^ were attributed to ν(N–H), ν(C=O), and ν(C=N) stretching vibrations, respectively (Figure S3). However, coordination of ligand **L1** with metal complexes was validated by observing a prominent shift in the diagnostic azomethine ν(C=N) stretching vibration peak at 1580–1570 cm^–1^.^26^ Moreover, a significant shift was observed in the ν(C=O) stretching vibrations to 1605 and 1598 cm^–1^ in complexes **1** and **2**, respectively, supporting the coordination of the carbonyl group (C=O) of hydrazone with the metal ion. In addition, the moderate-intensity peaks in the regions 550–540 and 500–480 cm^–1^ correspond to ν_M–O_ and ν_M–N_ stretching vibrations, respectively, thereby validating the coordination of O and N-atoms of **L1** with metal ions in complexes **1** and **2**.^27^

### 2.3 ^1^H and ^13^C NMR Studies

To ascertain the molecular structure of ligand **L1** and its diamagnetic complex **2**, ^1^H and ^13^C NMR studies were recorded in DMSO solution (Figures S4–S7). The ^1^H NMR spectrum of ligand **L1** and complex **2** displayed the characteristic singlet peaks centered at 11.5 and 11.8 ppm, which were attributed to the imine proton (NH) of the hydrazide unit (H_8_) and the indole ring (H_6_), respectively. However, in the ^1^H spectrum of complex **2**, the disappearance of the peak at 11.5 ppm suggested the coordination of ligand **L1** (possibly in the enolic form) via deprotonation.^28^ The diagnostic azomethine (−HC=N) singlet peak observed at 8.52 ppm (H1) in the spectrum of **L1** was found deshielded and shifted to 8.75 ppm in the spectrum complex **2**, thereby confirming its coordination with the Zn(II) ion. Moreover, the signature peaks of aromatic protons of **L1** and complex **2** were visible in the characteristic region of 6.8–8.2 ppm.^29^

The ^13^C NMR spectrum of **L1** revealed characteristic carbonyl (C9) and azomethine (C1) carbon peaks at 161 and 147 ppm, respectively. However, in complex **2**, these diagnostic peaks were found shifted and appeared at 177 and 145 ppm, respectively, thereby validating the coordination of carbonyl oxygen and azomethine nitrogen of **L1** with the Zn(II) ion.^28^ In addition, the corresponding spectra of **L1** and complex **2** displayed signature peaks of aromatic carbons in the region 110–140 ppm.

### 2.4 EPR Spectroscopy

The X-band EPR spectrum of complex **1** was recorded on a powdered sample at both room temperature (RT) and liquid nitrogen temperature (LNT) (Figure S8). The EPR spectrum revealed an isotropic signal, which signifies the paramagnetic nature of complex **1** due to the presence of five unpaired electrons. Moreover, the g values at RT and LNT were found to be 2.08 and 2.16, respectively, evidencing that the Mn ion in complex **1** is in the +2 oxidation state.^30^

### 2.5 Single-Crystal X-Ray Diffraction Studies

The structures of **L1** and complexes **1** and **2** were authenticated by single-crystal XRD studies. However, the single crystallographic data of **L1** was found similar to an earlier reported Schiff base by Xia et al. (Figure S9 and Table S1).^23^ Furthermore, all other bond parameters (bond angles and bond lengths) are also found within the range of reported hydrazide Schiff bases (Table S2).^28^

**Figure 1:**
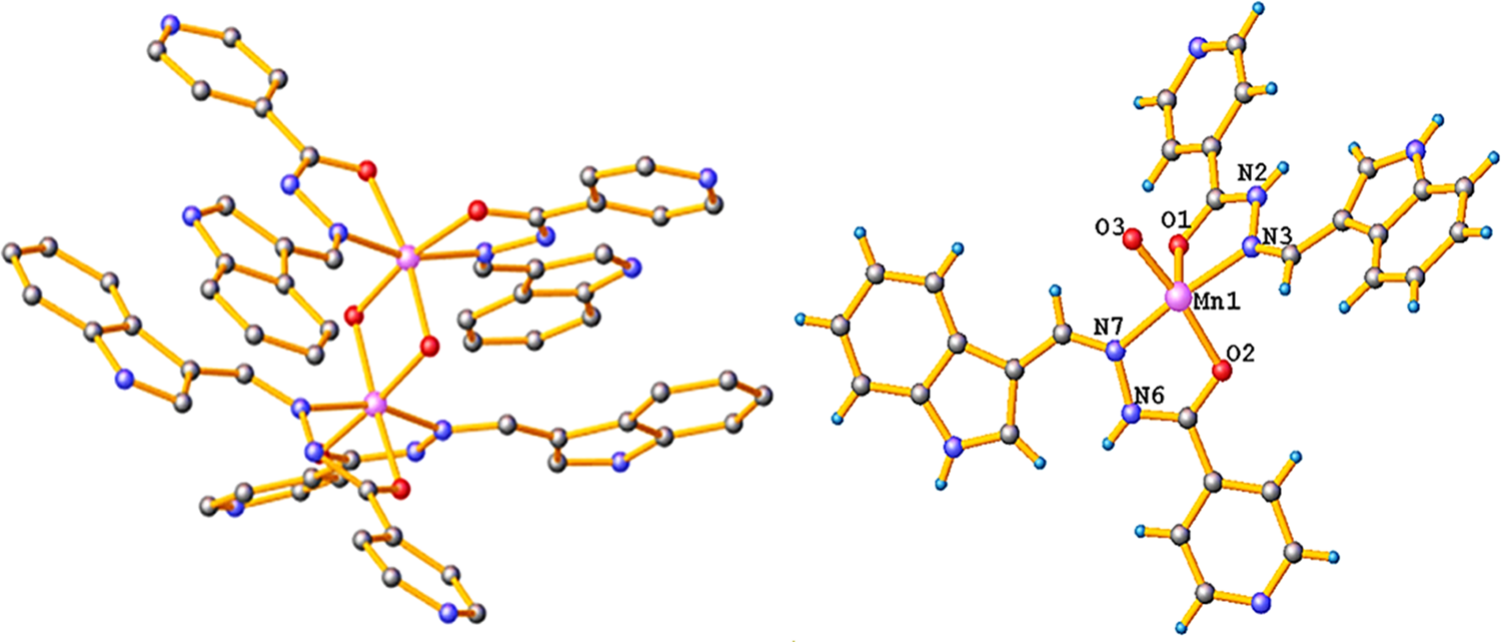
Single-crystal X-ray structure of complex **1** and its respective asymmetric unit with partial numbering. For clarity, water molecules and hydrogen atoms have been omitted.

Suitable crystals of metal complexes **1** and **2** for single-crystal XRD analysis were isolated from the corresponding reaction mixtures by a slow evaporation process. The s-XRD data revealed that complex **1** crystallized as a binuclear metal complex with a monoclinic system of space group *I*2/*a*. The cell parameters for complex **1** were measured as *a* = 15.0287(7) Å, *b* = 24.1629(8) Å, *c* = 16.1272(9) Å, and α = γ = 90° and β = 90.127°(2). In the crystal X-ray structure of complex **1**, both Mn(II) ions adopted a distorted octahedral geometry around metal ions in which each Mn(II) ion is coordinated to two bidentate Schiff base ligands via an azomethine N-atom and a carbonyl O-atom in a usual bidentate mode (Figure 1).^31^ The bond lengths, Mn–O (Mn1–O1 = 1.952(3) Å, Mn1–O2 = 1.969(4) Å) and Mn–N (Mn1–N3 = 1.987(4) Å, Mn1–N7 = 1.997(4) Å) bonds with two **L1** moieties, are in close approximation with reported Mn(II) Schiff base complexes.^32^ Furthermore, the other two coordination sites of each Mn(II) ion are completed via two μ–oxo bridgings with bond lengths Mn1–O3 = 1.819(4) and Mn1–O3 = 1.835(3), respectively.^33^ In addition, the respective bite angles (N3–Mn1–O1 and N7–Mn1–O2) formed by **L1** ligands with the center metal ion were measured as 78.76° (15) and 78.39° (16), respectively, which are fairly different from the regular pentagonal bond angle of 72°. Notably, all of the measured bond parameters were found within the range of reported binuclear Mn(II) complexes (Table S3).^34^

**Figure 2:**
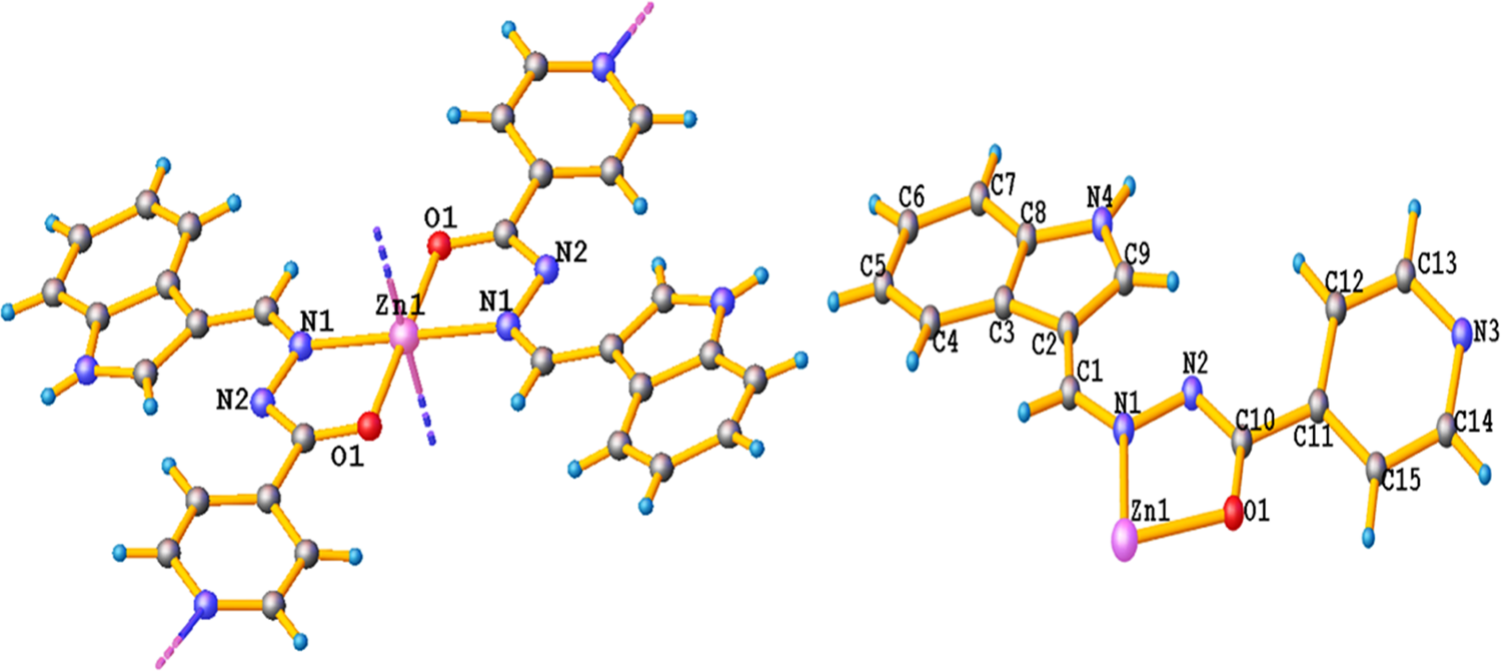
Single-crystal X-ray structure of complex **2** and its asymmetric unit with partial numbering of non-hydrogen atoms.

The single-crystal XRD data of complex **2** revealed that complex **2** crystallizes as a 2D polymer with a monoclinic crystal system of the *P*21/*n* space group. The lattice parameters were measured as *a* = 12.4274(10) Å, *b* = 13.3512(5) Å and *c* = 12.4653(10) Å, α = γ = 90° and β = 118.981° (10). In the crystal structure of complex **2**, each Zn(II) atom in one asymmetric unit is coordinated with **L1** via the N-atom of azomethine and the O-atom of the carbonyl group. The Zn–O (Zn1–O1 = 2.059 Å) bond lengths are identical, while the Zn–N (Zn1–N1 = 2.069 Å) bond lengths are also similar but slightly longer than the Zn–O bond distances (Table S4).^35^ In complex **2**, the Zn(II) ion adopted a distorted octahedral geometry in which the basal plane is occupied by N_2_O_2_ atoms of two **L1** ligands and is elongated in the axial direction by Zn–N (Zn1–N2 = 2.344 Å). The axial interactions between the Zn(II) atom of one asymmetric unit and the terminal N-atoms of the pyridine moiety of ligand **L1** with an adjacent molecule lead to an infinite 2D structure along the *x* and *y* crystallographic axes (Figures 2 and S10).^36^

### 2.6 Density Functional Theory (DFT) Studies

**Figure 3:**
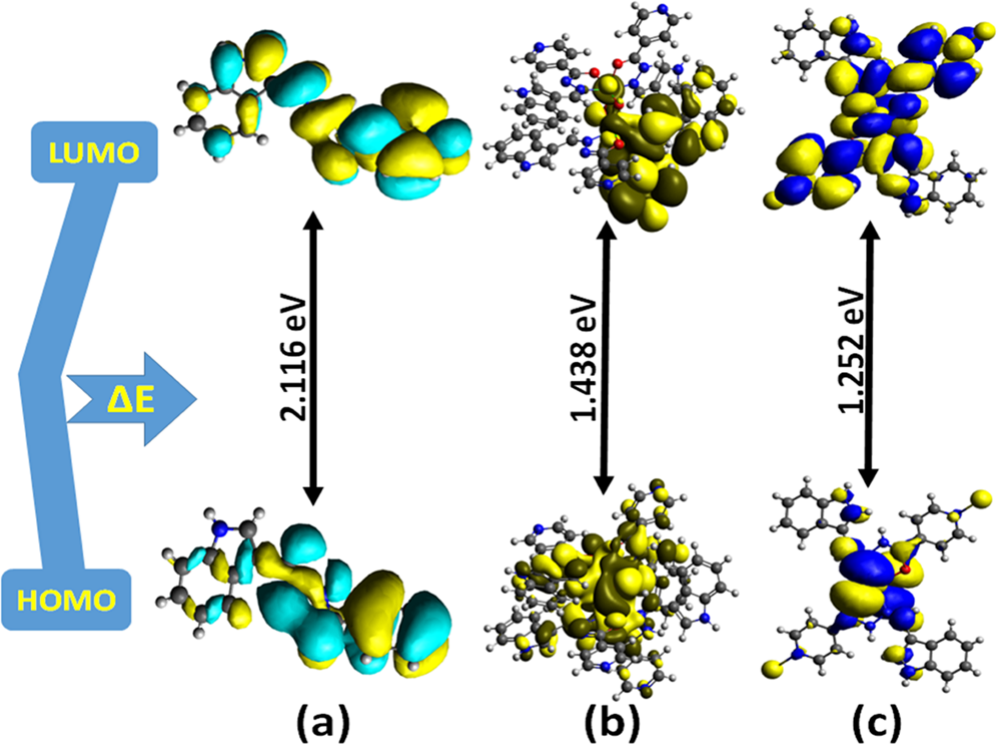
HOMO–LUMO energy gap of **L1** and complexes **1** and **2** (a–c) generated at the B3LYP hybrid functional.

DFT studies have been widely employed to predict the molecular geometry, relative conformational energy, electron affinity, and other thermodynamic parameters.^37^ From the calculated Frontier molecular orbitals (FMOs), it was evident that the imine and hydrazide moieties of **L1** are chemically more reactive because the electron density of HOMO and LUMO is mostly dispersed over the imine bond and partially localized on the isonicotinic hydrazide moiety of **L1** (Figure S11). However, in complexes **1** and **2**, the HOMO electron density was localized over the metal ions, while the electron density of LUMO was mostly delocalized over the ligands, with partial distribution on metal ions (Figures S12 and S13). The FMOs of **L1** and complexes **1** and **2** were further used to determine the chemical reactivity and kinetic stability by evaluating the universal indices of reactivity, viz., electronic potential (μ), global electrophilicity index (ω), electronegativity (χ), and chemical hardness (η), from their corresponding HOMO–LUMO energy gaps (Figure 3).^38^ From the values of these parameters described in Tables S5 and S6, it could be inferred that complex **2** is more electrophilic than complex **1** and **L1** and the chemical hardness increases on the order of **L1** > complex **2** > complex **1**.^39^ Moreover, the calculated bond parameters, viz., bond angles and bond lengths of ligand **L1** and complexes **1** and **2**, were found to be in good agreement with that of s-XRD data.

### 2.7 Hirshfeld Surface Analysis

**Figure 4:**
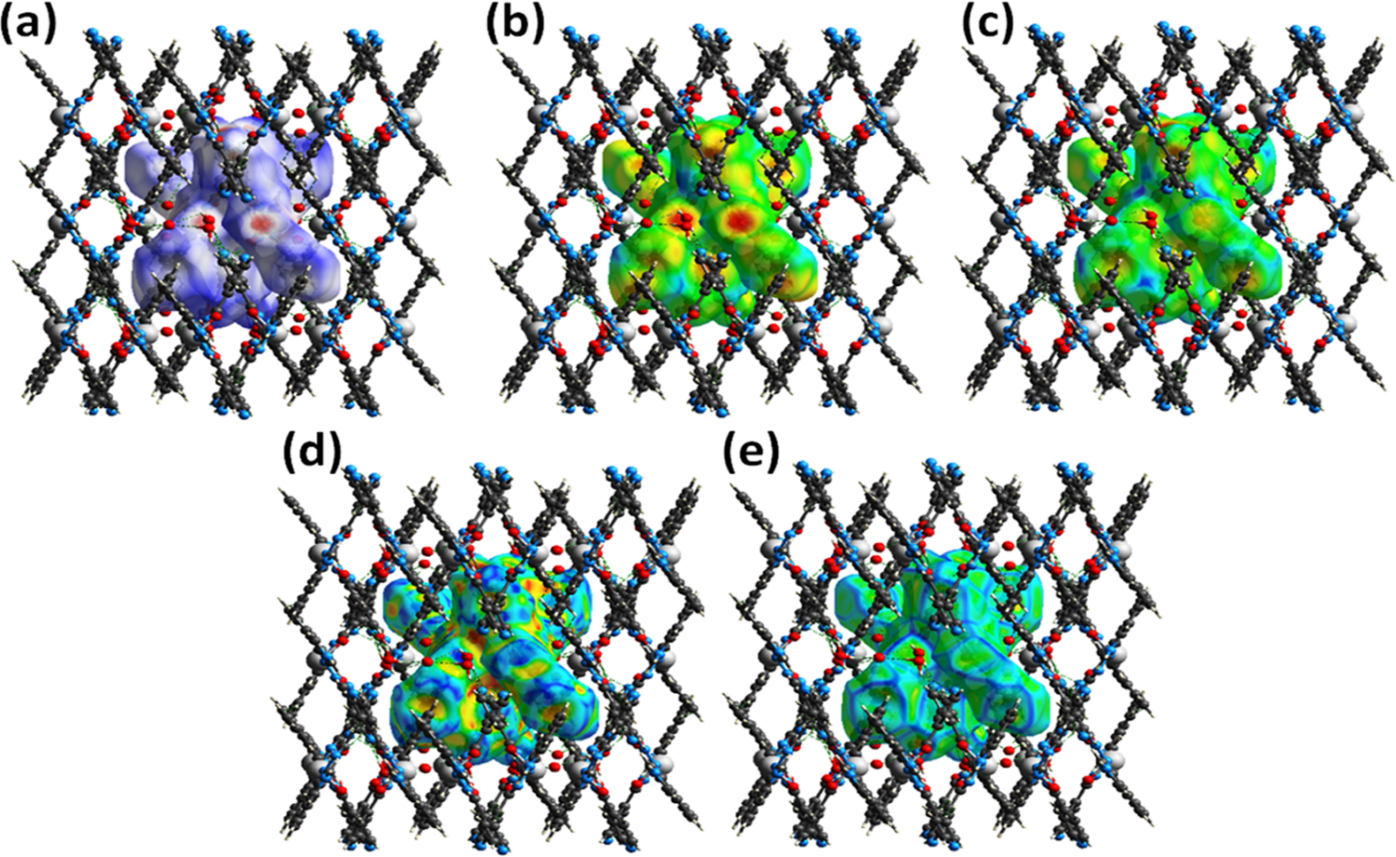
3D Hirshfeld surface mapping of binuclear complex **1**, (a) dnorm, (b) de, (c) di, (d) shape index, and (e) curvedness.

Hirshfeld surface analyses were employed to investigate the characteristics of various noncovalent interactions that occur within the crystal lattice (Figure 4). This study provides a detailed representation of these intermolecular interactions in terms of three-dimensional (3D) and two-dimensional (2D) fingerprint plots of crystal packing diagrams.^40^ An additional advantage of Hirshfeld surface analysis is that it allows one to decipher the molecular contacts in the form of 2D graphical plots between di and de. In our surface analysis, the 3D dnorm surfaces were plotted in the range of 0.5–1.5 Å, which portrays surfaces of red, blue, and white colors. Deep-red spherical regions are indicative of hydrogen-bonding interactions (O···H/N···H), blue color represents long intermolecular interactions, while white-colored regions correspond to weak intermolecular contacts whose radii are equal to van der Waals radii.^41^ Moreover, the adjoining red- and blue-colored regions on the 3D Hirshfeld surface of the shape index indicate that the molecules are connected to one another through π–π stacking interactions (Figures S14 and S15).^42^

**Figure 5:**
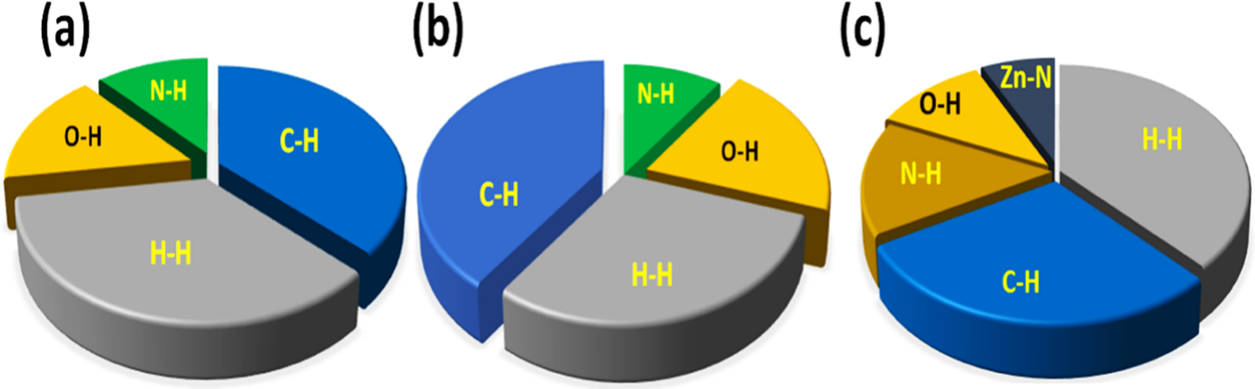
Pie-chart diagrams illustrating the percent contribution of various noncovalent interactions within the crystal lattices of (a) **L1**, (b) complex **1**, and (c) asymmetric unit of complex **2**.

Furthermore, 2D fingerprint plots offer a quantitative account of different intermolecular interactions, viz., O–H···H, N–H···H, and C–H···H, and essentially stabilize the crystal supramolecular structure.^40^ Interestingly, the H–H contacts showed the highest percentage contributions of 43.2 and 44.7%, out of all of the intermolecular interactions in complexes **1** and **2**, respectively, which substantiates the fact that the stability of crystals within the lattice is due to hydrogen-bonding interactions. The N···H and O···H interactions contribute equally to crystal packing in all compounds, which significantly increases the stability of their crystal lattices. Moreover, in complex **2**, sufficient contribution of Zn–N interactions (5.2%) was found due to its polymeric structure, while in the case of complex **1**, Mn–N interactions were found to be less than 1% (Figures 5 and S16).

## 3 *In Vitro* DNA Binding Studies

Most potential anticancer drugs exert their anticancer activity by targeting DNA via covalent or noncovalent binding interactions (intercalative, electrostatic, and groove binding).^43^ Thus, it was imperative to perform DNA binding interaction studies of the synthesized complexes with ct-DNA using multispectroscopic techniques to predict and validate their preferential binding mode.

### 3.1 Electronic Spectroscopy Titrations

**Figure 6:**
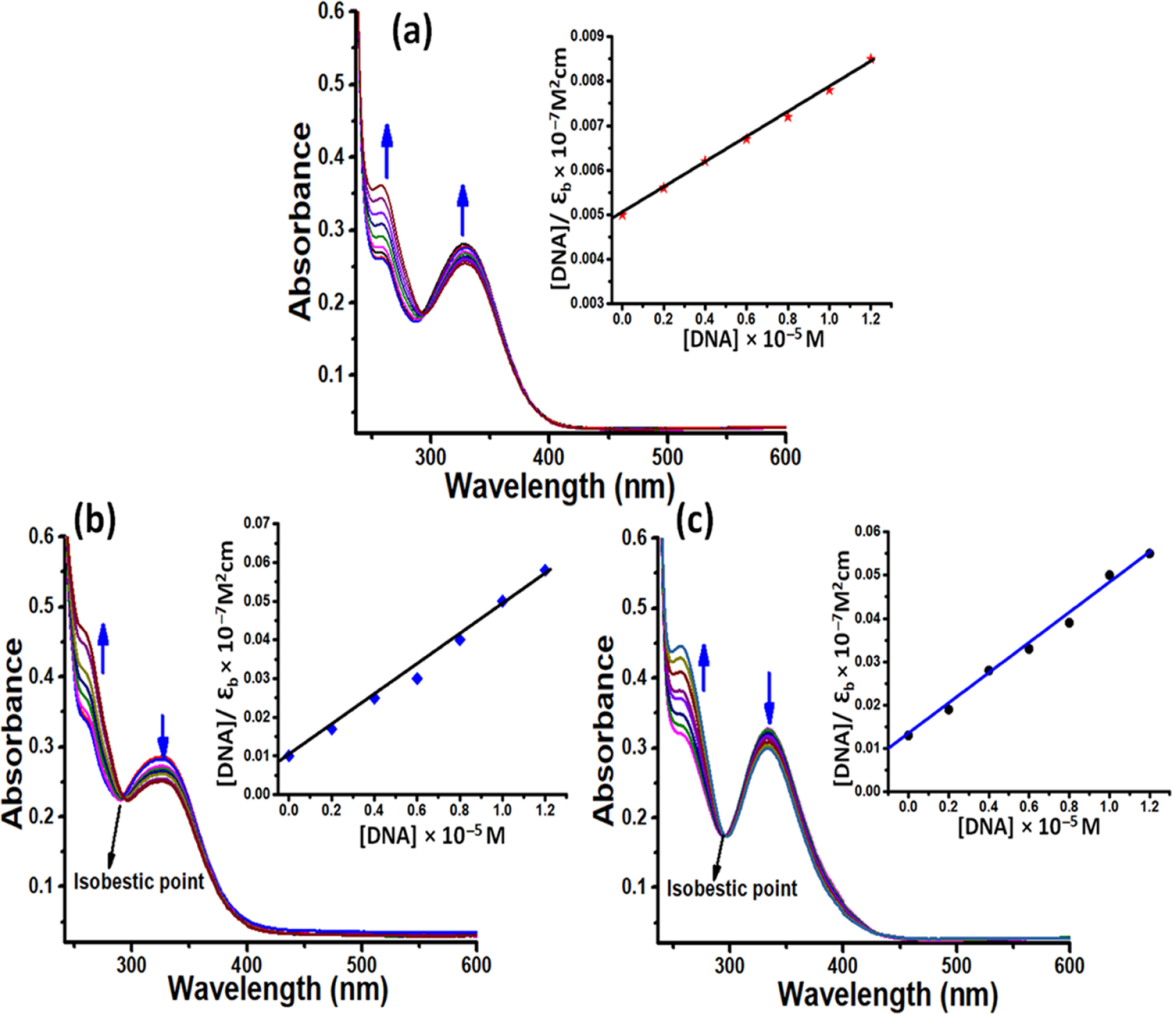
Absorption titration curves of **L1** and complexes **1** and **2** (a–c) at different concentrations of ct-DNA. Inset: Plots of [DNA]/ε_a_–ε_f_ (M^2^ cm) vs [DNA]. [DNA] = 0.1–0.8 × 10^–5^ M, [**L1**] = [Complexes **1** and **2**] = 0.5 × 10^–5^ M.

Electronic spectroscopy is one of the most frequently used techniques to investigate the nature and preferential mode of binding between metal complexes and nucleic acids. In our experiments, when the static concentration of **L1** (3 μM) and complexes **1** and **2** (5 μM) was titrated by adding aliquots of ct-DNA (1–8 μM), the spectral bands displayed hyperchromic change in the absorbance with a minor blue shift of 2–4 nm (Figure 6). The observed “hyperchromic effect” without any significant shift in the wavelength serves as the first possible evidence of the electrostatic mode of binding.^44^ In addition, the isosbestic point exhibited at ∼290 nm during the binding interaction indicates a single binding mode and the existence of an equilibrium between the DNA-bound and free form of metal complexes.^45^ Moreover, the stability of the binding interaction between synthesized compounds and DNA could be further contributed by forming a hydrogen bond between nitrogen and oxygen atoms of ligand **L1** and complexes **1** and **2** with the accessible nucleobases.

Furthermore, the magnitude of binding interaction of **L1** and complexes **1** and **2** was quantified by calculating the binding constant values using the Wolfe–Shimmer equation (as described in eq 5).^46^ The calculated binding constant values for **L1** and complexes **1** and **2** were found to be 8.89(±0.04) × 10^3^, 4.54(±0.17) × 10^4^, and 6.78(±0.11) × 10^4^ M^–1^, respectively, which substantiates the more avid binding of complex **2** than complexes **1** and **Ls1**. The better binding profile of complex **2** could be attributed to its polymeric nature and the specific topology, which facilitates multiple binding interactions with DNA.^47^

### 3.2 Fluorescence Spectroscopy

Fluorescence spectra of complexes **1** and **2** showed a considerable increase in intensity at ∼425 nm upon adding aliquots of ct-DNA (0.1–0.5 × 10^–5^ M) (Figure S17). The more nonpolar character of metal complexes in the presence of DNA could be the cause for the observed increase in the emission intensity, which also validated the substantial binding interaction between ct-DNA and metal complexes.^42^ Moreover, the binding constant values for complexes **1** and **2** were assessed from the Stern–Volmer equation (as described in eq 6) and were found to be 2.54 × 10^4^ and 4.29 × 10^4^ M^–1^, respectively.^48^ The obtained binding constant values substantiate the strong binding propensity of complex **2** and are consistent with the results of electronic spectroscopy.

### 3.3 Ethidium Bromide (EB) Assay

EB competitive analysis was used to assess the preferential mode of interaction between ct-DNA and metal complexes. EB is a conjugate planar cationic dye and exhibits a weak emission in Tris-HCl buffer; however, it displays an intense luminescence in addition with DNA due to its strong insertion within DNA base pairs at 580 nm.^49^ In our experimental study, upon adding aliquots of complexes **1** and **2** to a static concentration of the EB-DNA system, moderate quenching was observed, which suggests that complexes **1** and **2** have a poor ability to displace EB within the DNA base pairs and hence ruled out the intercalative mode of binding (Figure S18). These results further validated that complexes **1** and **2** possibly interact with DNA via the electrostatic mode of interaction. According to the Stern–Volmer equation, *K*_sv_ values were determined for complexes **1** and **2** (as described in eq 6) and the values were found to be 4.69(±0.14) × 10^4^ and 9.43(±0.17) × 10^4^ M^–1^.^50^ Moreover, it is evidenced from respective *K*_sv_ values that complex **2** showed a better binding efficiency toward ct-DNA than complex **1** and corroborates well with the results of electronic spectroscopy.

### 3.4 Electrochemical Studies

Electrochemical studies of complexes **1** and **2** were performed at room temperature in the scan range of −1.5 to +1.5 V in a Tris-HCl buffer solution of pH 7.3. These studies were used to examine the electrochemical behavior of metal complexes and also to further complement spectroscopic studies (Figure S19).^51^ It is documented that a positive shift in the electrode potential signifies intercalative mode of interaction while negative electrode potential shift validates groove binding or electrostatic mode of interaction.^52^ In our experimental studies, the cyclic voltammogram curves of complexes **1** and **2** displayed a quasi-reversible redox peak with an anodic potential in the range of 595–650 mV, while a cathodic peak potential was observed in the range of 620–614 mV corresponding to redox couples of Mn(II)/Mn(I) and Zn(II)/Zn(I), respectively.^53^ The differences between cathodic and anodic peak potentials for complexes **1** and **2** were found to be −24 and −36 mV, respectively (Table S7). Moreover, the ratio of anodic and cathodic currents in both complexes is approximately unity, which indicates a single electron-transfer mechanism in complexes **1** and **2**. However, upon gradual additions of ct-DNA, no new redox peak was observed in the voltammogram of complexes **1** and **2**, while a considerable decrease in current was observed, which suggested that metal complexes **1** and **2** have considerable binding interaction with ct-DNA. The significant reduction in the magnitude of current is due to the equilibrium mixing of the DNA-bound and free form of the metal complexes.^54^ Moreover, the significant negative shift in anodic and cathodic electrode potentials upon increasing aliquots of ct-DNA to the constant concentration of metal complexes further validated the electrostatic mode of interactions between complexes **1** and **2** and ct-DNA.^51^

### 3.5 Circular Dichroism (CD)

CD studies were employed to examine the structural and morphological changes in the secondary structure of B-DNA, upon interaction of metal complexes with DNA. Native DNA existing in the B-DNA form displayed two distinctive signature bands at 275 nm (positive band) and 245 nm (negative band) attributed to the right-handed helicity and base stacking, respectively.^55^ It is documented that upon interaction of complexes via the intercalative mode, the intensity of both signature bands is enhanced significantly, while in the case of a simple groove or electrostatic binding mode of interaction, minor or no change is observed. In our CD experiments, when complexes **1** and **2** were added to ct-DNA, the intensity of the signature band at 275 nm increased significantly, while at 245 nm, a decrease in intensity was observed due to DNA helix unwinding, which eventually resulted in a loss of DNA helicity, thereby validating the electrostatic mode of interaction.^56^ However, alterations in the CD spectrum clearly indicated the noncovalent contacts between the metal complexes and ct-DNA, which consequently ruled out the intercalative mode of binding (Figure S20).

## 4 Protein Binding Studies

Serum albumin proteins play a key role in effectively delivering the therapeutic drugs to their respective biological targets.^49^ The apparent stability and therapeutic efficacy of metallodrugs are often determined by their interaction with serum albumin proteins.^57^ To explore the binding potential and a consequent therapeutic effect of synthesized metal complexes, interaction studies with the BSA protein were performed by employing spectroscopic methods.

### 4.1 Absorption Titration Studies

**Figure 7:**
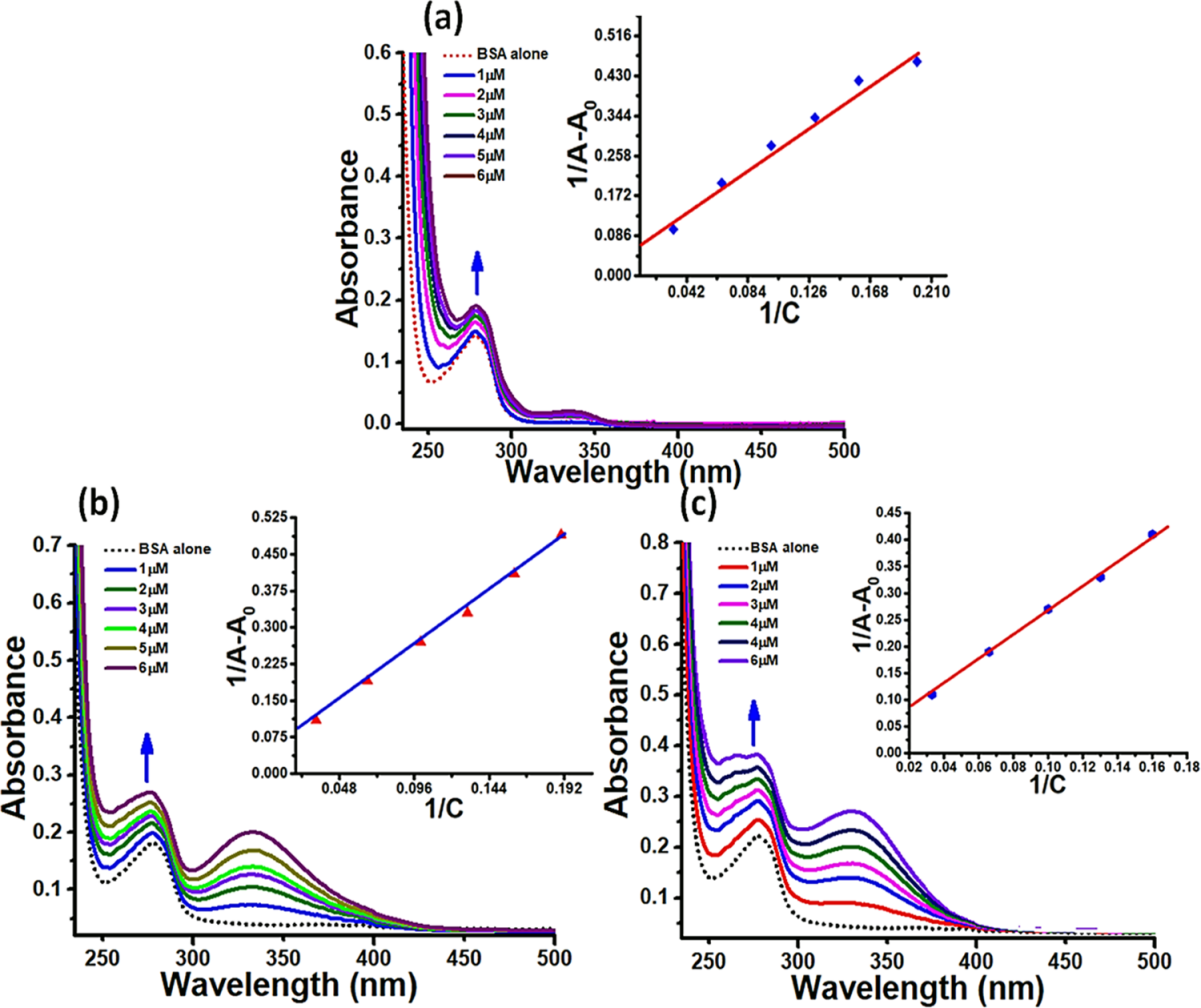
Absorption titration curves of BSA (dotted line) upon incremental additions of **L1** and complexes **1** and **2** (a–c). Arrows represent the corresponding change in spectra upon increasing concentrations of **L1** and complexes **1** and **2** (0.1–0.7 × 10^–5^ M).

In the UV–vis spectrum of BSA, a characteristic band was displayed at 280 nm assigned to π–π* transitions of amino acid residues, viz., Trp-135 and Trp-214.^58^ Upon the gradual addition of ligand **L1** and complexes **1** and **2** (1–7 μM) to a static concentration of BSA (4 μM), a progressive increase in absorbance (hyperchromism) was observed at λ_max_ = 280 nm (Figure 7). This observed hyperchromic change in the protein absorption band of BSA suggested the noncovalent binding mode of interaction between the ligand (**L1**) and complexes **1** and **2** within the hydrophobic environment of BSA. However, more alterations in the BSA conformation in the case of complex **2** were inferred by observing shifts in the characteristic protein band and percent hyperchromism (29% for **L1**, 32% for complex **1**, and 47% for complex **2**). Binding constant (*K*_b_) values were calculated (using eq 8) to further determine the extent of binding strength and were found to be 3.83(±0.25) × 10^3^, 4.18(±0.13) × 10^4^, and 9.78(±0.22) × 10^4^ M^–1^ for **L1** and complexes **1** and **2**, respectively. The higher *K*_b_ value of complex **2** validates its more efficient binding toward the BSA protein in contrast to **L1** and complex **1**.

### 4.2 Fluorescence Quenching Studies

Interaction studies of complexes **1** and **2** were further performed by observing the change in the emission spectra of BSA upon adding aliquots of complexes **1** and **2**. The tryptophan residue (Trp-214) emission is primarily responsible for the strong intrinsic fluorescence of the BSA protein, thereby making it subtle to changes in the microenvironment of a protein fluorophore.^59^ From the recorded emission spectra, a regular decrease in fluorescence maxima at 342 nm was observed upon cumulative addition of complexes **1** and **2** (0.1–0.5 μM), indicating significant binding interaction of metal complexes **1** and **2** with BSA. In addition, significant quenching effect was observed in the emission spectra of complex **2**, thereby suggesting its stronger binding affinity toward the BSA. Moreover, the higher binding efficiency of complex **2** was further quantified by calculating the *K*_*b*_ values of complexes **1** and **2** using the Stern–Volmer equation (as described in eq 6), and the values were found to be 2.18 × 10^4^ and 6.78 × 10^4^ M^–1^, respectively.

### 4.3 Circular Dichroism

CD spectra of native BSA exhibited two distinctive bands in the far-UV region at 222 and 208 nm, which are attributed to π–π* and n−π* transitions of the peptide bond, respectively (Figure S22).^60^ However, a considerable increase in intensity was observed after adding aliquots of complexes **1** and **2** to the constant concentration of BSA, validating the significant interaction of BSA with metal complexes. However, upon addition of complexes **1** and **2**, no obvious shift in the position of the characteristic BSA bands was observed, validating that the structure of the BSA protein was primarily in an α helical shape.^61^

## 5 Molecular Docking

**Figure 8:**
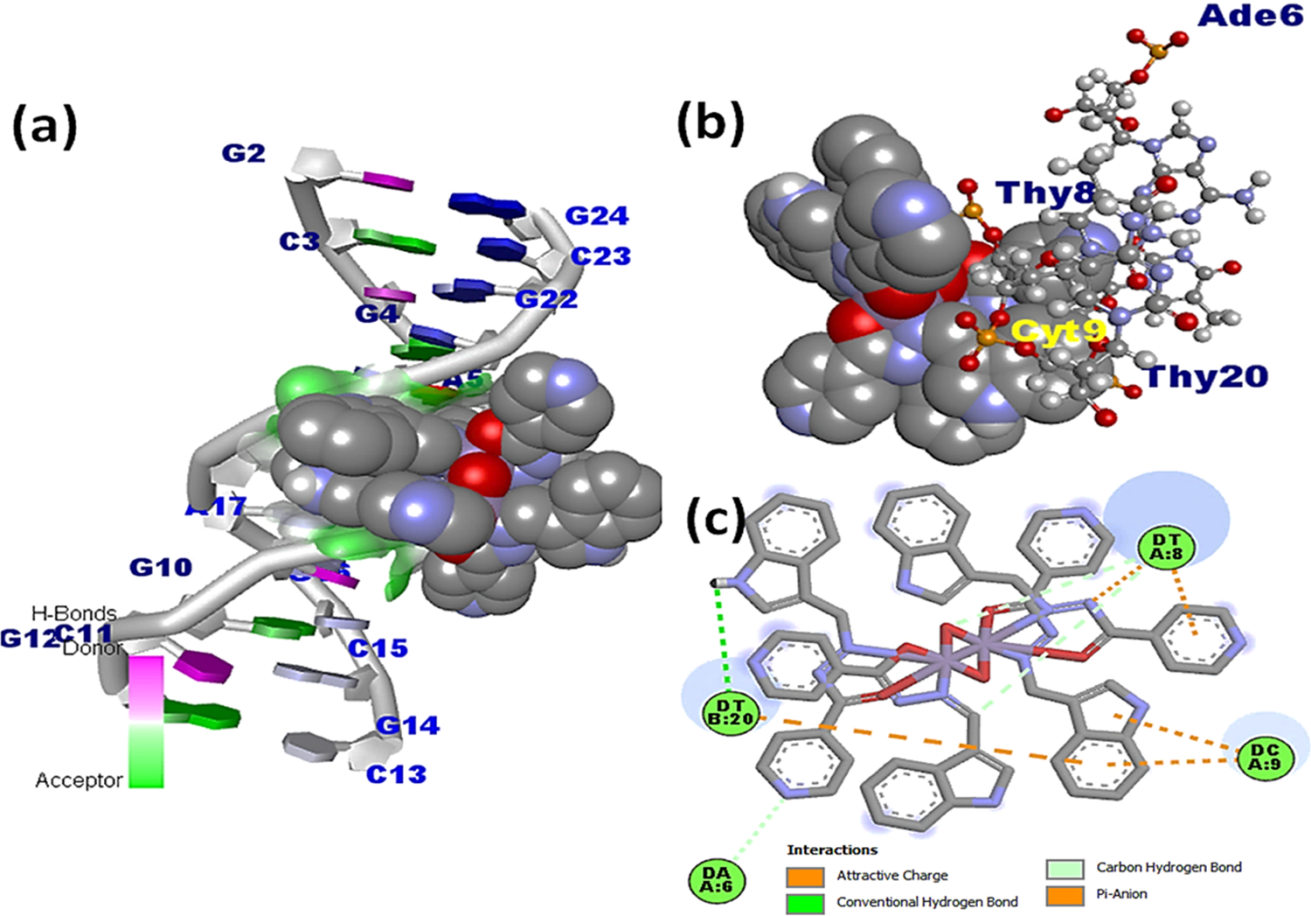
Docked model of complex **1** with DNA illustrating (a) complex **1** fitted within the G–C base pair region of DNA; (b) interaction of complex **1** with different nucleotides in 3D view; and (c) ligand nucleotide interaction in 2D view.

Molecular docking studies were employed to comprehend the drug–macromolecular interactions and predict the specific binding sites that are available at the molecule target.^62^ The docked structure of ligand **L1** with DNA revealed that **L1** was located in the vicinity of Ade5, Ade6, Gua22, Cyt21, and Thy20 base pairs, and notably, hydrogen bonds were formed between the nitrogen and oxygen atoms of the hydrazine and indole moieties with the hydrogen atoms of Ade5 (2.81 Å) and Cyt21 (2.54 Å), respectively (Figure S23). However, in the docked models of complexes **1** and **2**, it was observed that the complexes preferred an A–T-rich region of the DNA (Figures 8 and S24). Since electronegative A–T base pair sequences are smaller than G–C base pair sequences, they provide better fitting for small molecules and generally provide superior van der Waals interactions than G–C base pairs.^63^ Complex **1** was found in the close vicinity of Ade5, Ade6, Gua4, Gua16, Cyt3, and Thy19 base pairs, while complex **2** was found closer to Ade6, Cyt9, Thy8, and Thy20 base pairs. The stability of the complex DNA docked model may also be influenced by the hydrogen bonding between the N-atom of the hydrazide and indole moiety with the hydrogen atoms of the minor groove base pairs Thy19 (2.150 Å), Ade6 (2.158 Å), Cyt9 (2.945 Å), and Thy8 (3.679 Å). The binding strengths of **L1** and complexes **1** and **2** were quantified by their respective free binding energy values, which were found to be −8.72, −7.1, and −6.8 kcal mol^–1^. The more efficient binding strength of complex **2** than complex **1** and **L1** is indicated by its less negative binding energy value.

**Figure 9:**
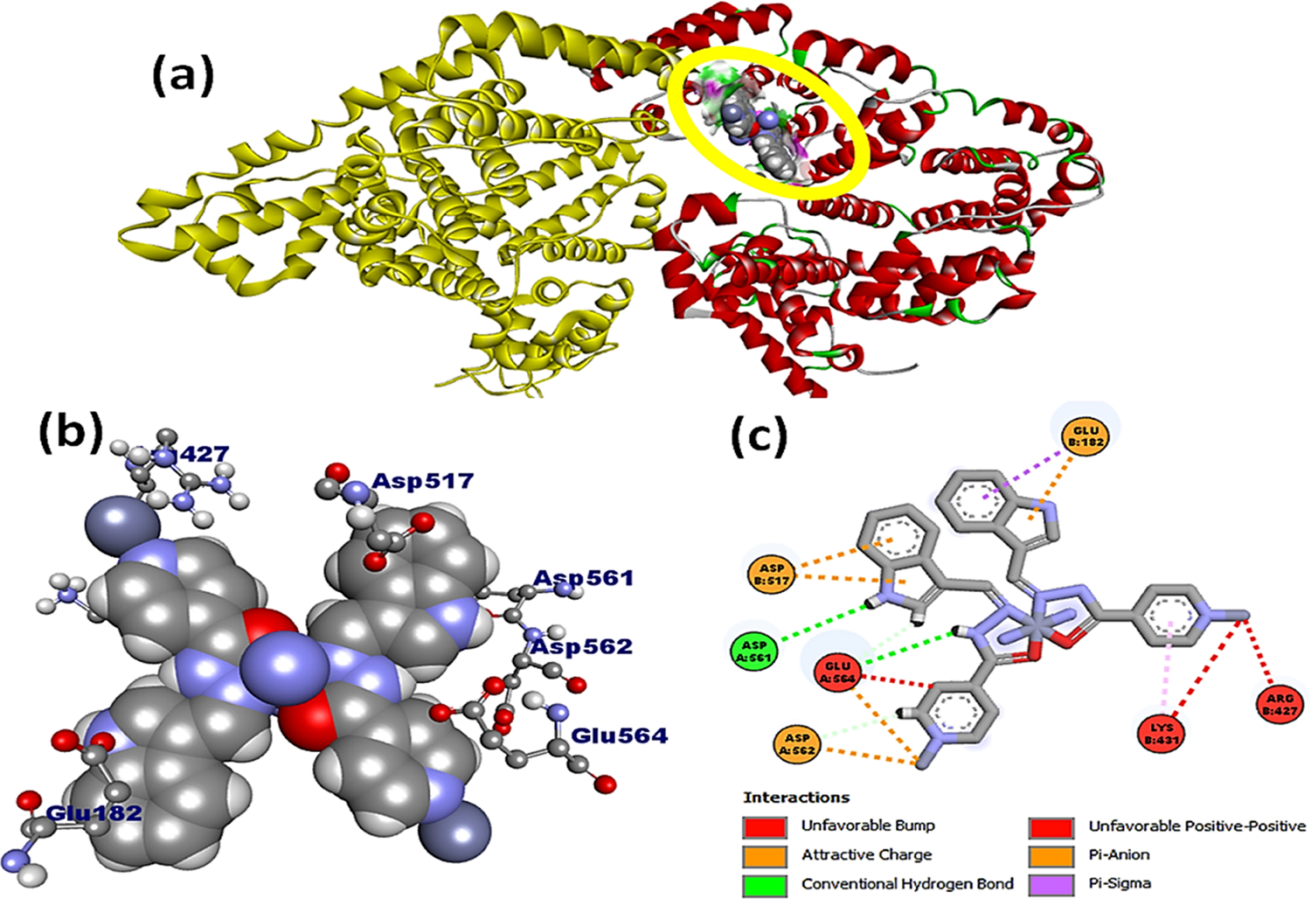
(a) Docked model of complex **2** within subdomain IIA of BSA; (b) interaction of complex **2** with various amino acid residues displayed in 3D view; and (c) interactions of complex **2** with amino acid residues in 2D view.

Complexes **1** and **2** were further docked with the BSA protein, and the resulting docked model showed that **L1** and complexes **1** and **2** were localized in subdomain IIA of BSA (Figures 9, S25, and S26). Complex **1** exhibited preferential binding affinity near the amino acid residues, viz., Asp 172, Ala 176, Ala 510, Glu 182, Glu 664, His 603, Leu 176, Pro 117, and Pro 173, and is located in the hydrophobic binding pocket of BSA. Interestingly, complex **2** was also found in a similar binding site within the close proximity of amino acid residues, viz., Asp 617, Asp 561, Asp 582, Arg 427, Glu 182, Glu 664, and Lys 431. The complex **1**–BSA docked model was typically stabilized by hydrogen bonding with adjoining amino acid residues of the specific binding site, viz., Asp 561 (2.348 Å) and Glu 182 (2.985 Å), whereas the complex **2**–BSA docked model forms hydrogen bonds with Glu 664 (2.469 Å) and Glu 182 (2.784 Å) amino acid residues with the nitrogen atom of the indole moiety. Moreover, the existence of a strong aquaphobic interaction between metal complexes and BSA correlated well with the results obtained from the various biophysical investigations. Free binding energies for **L1** and complexes **1** and **2** with BSA were determined to be −7.9, −7.4, and −6.9 kcal mol^–1^, respectively. These values further substantiate that complex **2** has a better binding affinity than **L1** and complex **1** and are consistent with the spectroscopic results.

## 6 Antioxidant Activity

**Figure 10:**
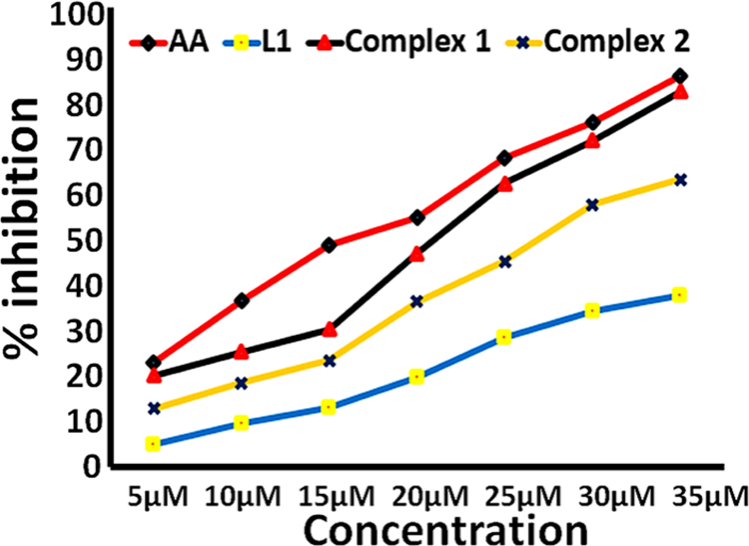
Free radical scavenging activity of **L1** and complexes **1** and **2** in comparison with ascorbic acid (as standard).

Oxidative stress involves the persistent accumulation of oxygen-based free radicals (superoxide, peroxide, hydroxyl, etc.) and eventually leads to several diseased conditions.^64^ These free radicals can be effectively scavenged by various redox active complexes before affecting the electron-rich biomolecules. We have validated the antioxidant activity of complexes **1** and **2** via the DPPH free radical assay by employing electronic spectroscopy.^65^ Upon progressive additions of **L1** and complexes **1** and **2** (5–35 μM) to a fixed concentration of DPPH (2 μM), an obvious decrease in the absorbance intensity at 517 nm was observed. However, in the case of complex **1**, a prominent decrease in absorbance (38%) indicated its more efficient antioxidant potential against the DPPH free radical than **L1** and complex **2**. Furthermore, the antioxidant nature of complexes **1** and **2** was measured by calculating IC_50_ values and the corresponding percent radical scavenging activity values (Table S8). The calculated results were found in the following decreasing order: AA (80.59%) > complex **1** (78.14%) > complex **2** (61.44%) > **L1** (49.66%) (Figure 10), which further validates that complex **1** has a significantly higher antioxidant nature than complex **2** and the free ligand.^66^

## 7 Antibacterial Activity

The antibacterial studies of **L1** and complexes **1** and **2** were performed against four bacterial strains, viz., *Staphylococcus aureus* and *Bacillus subtilis* (Gram-positive bacteria) and *Escherichia coli* and *Pseudomonas aeruginosa* (Gram-negative bacteria). In our experiments, minimum inhibitory concentration (MIC) and the zone of inhibition values were used to measure the antibacterial activities of **L1** and complexes **1** and **2** (Figure S27).^67^ Our results revealed that complexes **1** and **2** showed significant antibacterial activity against Gram-positive bacteria, which was evidenced from the maximum zone of inhibition and their low MIC values (Tables S9 and S10).^68^ However, in ligand **L1**, moderate bactericidal action was observed at the same concentration. The better antibacterial activity of metal complexes against Gram-positive bacteria could be ascribed to the structural alteration of ligand **L1** upon complexation with the metal ions.^69^ Moreover, the higher antibacterial activity of complexes **1** and **2** can be explained with respect to the overtone theory, according to which the ligand metal coordination results in a significant increase of the lipophilicity of metal complexes and a consequently high cellular permeability.^70^ The facile transport of metal complexes across the bacterial cell membrane causes their potential to interfere with the cellular metabolism and organelles with an eventual bactericidal effect.^71^ In addition, the antibacterial activity of complexes **1** and **2** was compared with similar reported Mn(II)/Zn(II) aroyl-hydrazone complexes, and the results were found to be consistent with earlier reported results.^68,72^

## 8 *In Vitro* Cytotoxic Activity

**Figure 11:**
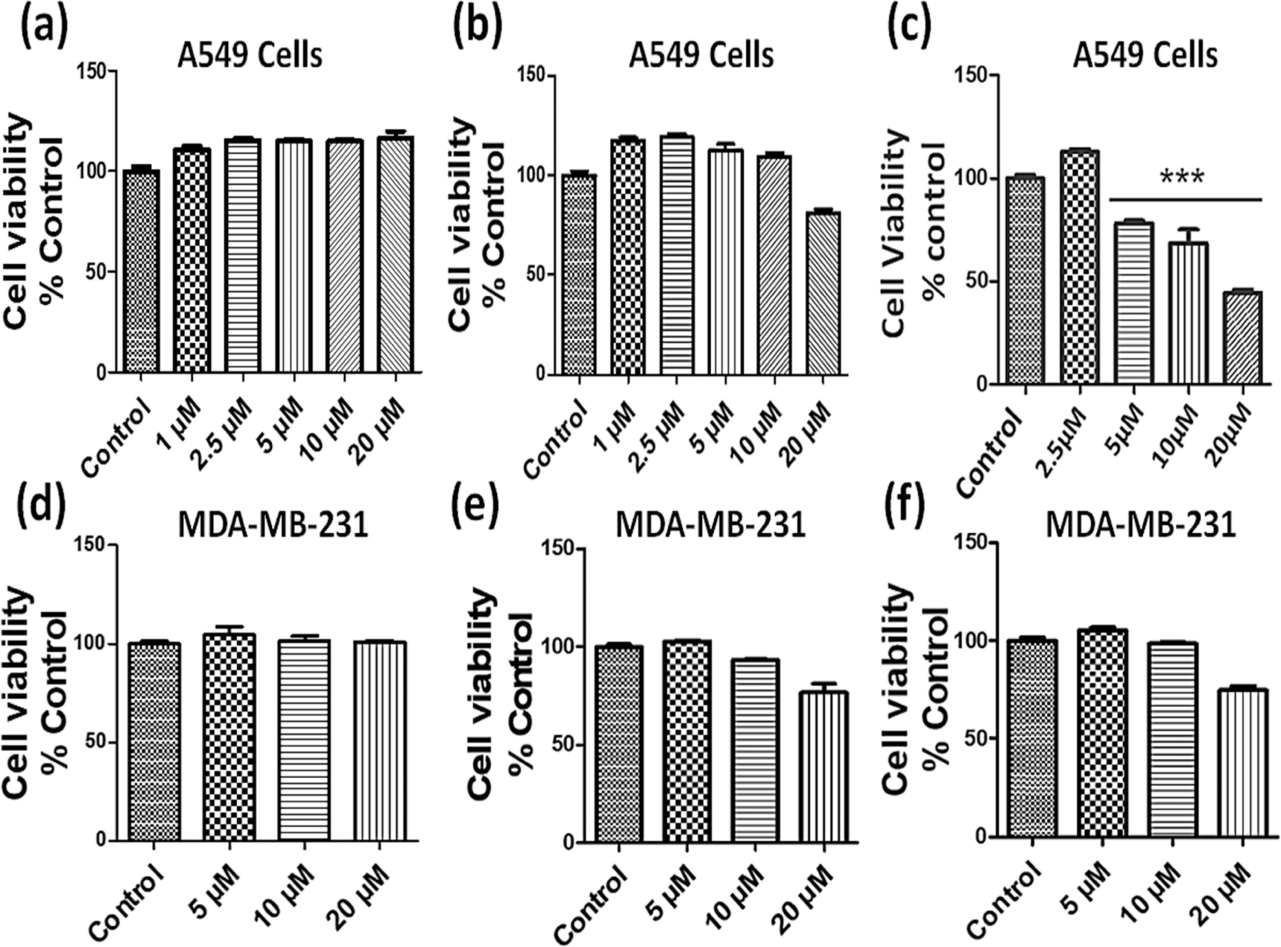
*In vitro* cell viability of A549 and MDA-MB-231 cancer cells when treated with different concentrations of **L1** (a, d), complex **1** (b, e), and complex **2** (c, f). Data represent mean ± SEM of three independent trials (*n* ≥ 3).

*In vitro* cytotoxic evaluation of **L1** and complexes **1** and **2** was carried out against A549 (lung) and MDA-MB-231 (triple negative breast) cancer cell lines using the MTT assay.^73^ In our experiments, the cytotoxic effect of **L1** and complexes **1** and **2** was evaluated against the viability of these cells at different concentrations after an exposure of 48 h (Figure 11). The results of the percent growth inhibition experiments revealed that **L1** and complex **1** exhibit poor cytotoxic activity as compared to that of the control (DMSO) and complex **2** (Tables S11 and S12). However, complex **2** demonstrated selective cytotoxic activity against the A549 cell line with a fairly low IC_50_ value of 17.54 μM (Figure 11). The selective cytotoxic activity of complex **2** against A549 cancer cells may be attributed to its polymeric structure, which facilitates multiple binding interactions between the target and complex **2**.^47,74^ Additionally, the cytotoxicity of polymeric complex **2** against the A549 cancer cell line was compared with previously reported similar zinc hydrazone Schiff base complexes, and cisplatin. Complex **2** demonstrated better cytotoxicity against the tested cancer cell line in comparison to the previously reported zinc hydrazone Schiff base complexes and cisplatin, which was evidenced from their IC_50_ value (Table S13).^75,76^ Therefore, it may be speculated that complex **2** is a promising chemotherapeutic agent not only for better cytotoxic efficiency but also for its low toxicity.

## 9 Experimental Section

### 9.1 Materials, Reagents, and Instrumentation

The commercially obtained solvents and reagents are used without any additional purification. Indole-3-carboxaldehyde, isonicotinic hydrazide, calf thymus deoxyribonucleic acid (ct-DNA), 2,2-diphenyl picrylhydrazyl (DPPH), Tris-buffer (Tris-(hydroxymethyl)aminomethane), bovine serum albumin (BSA), manganese acetate, and zinc acetate were bought from commercial sources (Sigma-Aldrich and Alfa Aesar) and were utilized as received.

A Perkin-Elmer 240C elemental analyzer was utilized for carbon, hydrogen, and oxygen elemental analyses. Perkin-Elmer was used to perform Fourier transform infrared (FTIR) of **L1** and complexes **1** and **2** in the mid-IR range of 400–4000 cm^–1^. Electron paramagnetic resonance (EPR) spectra of complex **1** were recorded by a Varian E112 EPR spectrometer operating at 9.5 GHz in the X-band. Using a LABMAN conductivity meter of model LMCM-20, measurements of the molar conductivity of complexes **1** and **2** were performed. Quartz cuvettes with a 1 cm path length were used to perform UV–vis spectra on the Perkin-Elmer Lambda 35, and data were recorded in λ_max_ (nm). A spectrofluorophotometer (Shimadzu RF-5301PC) was employed throughout the experiments for emission studies. For the purpose of measuring circular dichroism, a Jasco J-815-CD spectropolarimeter equipped with a Peltier temperature control mechanism was employed. Using the Evans method and magnetic susceptibility balance (Sherwood Scientific), magnetic studies were performed. On a JEOL resonance model JNM-ECZ400S/L1 spectrometer operating at 400 MHz, NMR experiments were carried out. At the electrochemical analyzer for the CH instrument, cyclic voltammetry was performed in a single compartment cell with a three-electrode setup; all electrochemical tests were carried out. Pt wire, Ag/Ag^+^, and Pt spheres were used as working, reference, and auxiliary electrodes in this investigation, respectively. Throughout the experiment, 0.4 M potassium nitrate in Milli-Q water was employed as the supporting electrolyte. The cathodic (*E*_pc_) and anodic (*E*_pa_) peak potential values for the synthesized complexes were used to compute the half-wave redox potential (*E*_1/2_) using the equation (*E*_pa_ + *E*_pc_)/2.

### 9.2 Synthesis of Ligand **L1**

An equimolar methanolic solution of indole-3-carboxaldehyde (2 mM, 0.290 g) was added dropwise to the methanolic solution of isonicotinic hydrazide (2 mM, 0.275 g) under continuous stirring and reflux, resulting in the formation of the aroyl-hydrazone Schiff base ligand (**L1**) as a bright-yellow solid. The resulting yellow solid was filtered off and washed with cold methanol. After 2 days of evaporation, yellow-colored needle-shaped crystals suitable for single-XRD studies were obtained from the filtrate.

Yield 72%, MP: 145 °C, Anal. calc. for C_15_H_12_N_4_O (%): calc. C, 68.44; H, 4.56; N, 21.29; found: C, 68.08; H, 4.37; N, 21.34: UV–vis (1 × 10^–3^ M, λ_max_ nm) in DMSO: 263 (π–π*), 327 (n−π*): FTIR data on KBr pellet (ν/cm^–1^): ν(N–H) 3396 cm^–1^; ν(C=O) 1675 (s); ν(C=N) 1602 (s); ν(C=C aromatic) 1551 (m); ν(C–N pyridine) 1297 (m); ^1^H NMR (DMSO-*d*_6_, 400 MHz), δ (ppm): 3.20 (s, 6H, DMSO), 6.8 (d, 1H, ArH), 7.10 (m, 1H, ArH), 7.41 (s, 1H, indole-H), 7.83 (m, 2H, pyridine-H), 8.21 (d, 1H, ArH), 8.52 (s, 1H, azomethine), 8.74 (m, 2H, pyridine-H), 11.61 (s, 1H, NH), 11.80 (s, 1H, indole-NH): ^13^C NMR (DMSO-*d*_6_, 400 MHz), δ (ppm): 111.04 (indole-C2), 112 (Ar-C6), 120–125 (Ar-C3, C4, C5), 131 (indole C7 and C8), 138 (pyridine-C11), 142.14 (pyridine-C10), 147 (azomethine-C1), 151.23 (pyridine-C12), 161.04 (carbonyl-C9); CCDC: 2191272.

### 9.3 Synthesis of Complexes **1** and **2**

The synthesis of complexes **1** and **2** was accomplished by adding a methanolic solution of manganese acetate tetrahydrate (1 mM, 0.245 g)/zinc acetate dihydrate (1 mM, 0.221 g) to the Schiff base ligand solution, **L1** (2 mM, 0.264 g), under reflux conditions, which yielded complexes **1** and **2** as dark-black and orange-colored solutions. The resulting reaction mixtures of complexes **1** and **2** were filtered and left for crystallization by a slow evaporation process at room temperature.

#### 9.3.1 Complex **1**

Yield 78%, MP 290 °C, Anal. calc. for C_60_H_56_Mn_2_N_16_O_12_ (%), calc. C, 55.29; H, 4.30; N, 17.20; found: C, 56.15; H, 4.38; N, 17.61: UV–vis (1 × 10^–3^ M, λ_max_ nm): 261 (π–π*), 323 (n−π*); FTIR data on KBr pellet (ν/cm^–1^): ν(N–H) 3258 (m); ν(C=N) 1593 (s); ν(C=C) 1548 (m); υ(C–N) 1227; Λ_M_ (Ω^–1^ mol^–1^ cm^2^) in DMSO: 13.15; μ_eff_ = 5.49 BM; CCDC: 2191274.

#### 9.3.2 Complex **2**

Yield 72%, MP 275 °C, Anal. calc. for C_34_H_38_ZnN_4_O_4_ (%), calc. C, 63.40; H, 6.32; N, 9.24; found: C, 63.44; H, 6.34; N, 9.25: UV–vis (1 × 10^–3^ M, λ_max_ nm) in DMSO: 265 (π–π*), 335 (n−π*); FTIR data on KBr pellet (ν/cm^–1^): ν(N–H) 3421 cm^–1^; ν(C=O) 1657 (s); ν(C=N) 1597 (s); ν(C=C aromatic) 1521 (m); ν(C–N pyridine) 1230 (m); ^1^H NMR (DMSO-*d*_6_, 400 MHz), δ (ppm): 3.20 (s, 6H, DMSO), 7.2 (m, 2H, ArH), 7.5 (d, 1H, ArH), 7.8 (s, 1H, indole-H), 8.1 (m, 2H, pyridine-H), 8.2 (d, 1H, ArH), 8.75 (s, 1H, azomethine-H), 8.96 (m, 2H, pyridine-H), 11.9 (s, 1H, indole-NH), ^13^C NMR (DMSO-*d*_6_, 400 MHz), δ (ppm): 108.04 (Ar-C), 113 (Ar-C), 118 (Ar-C), 118 (pyridine-C5), 122(C3, C4), 128 (C7, C8), 135 (C11), 137 (C10), 145 (C1), 150 (C12), 177 (C9), Λ_M_ (Ω^–1^ mol^–1^ cm^2^) in DMSO: 11.83; CCDC: 2191272.

### 9.4 Single-Crystal XRD Description

An XtaLAB Synergy-1 diffractometer with a dual microfocus, sourced from Rigaku corporation, Japan, was used to collect the crystallographic details of complexes **1** and **2** at room temperature by employing monochromated (Mo Kα) radiation (0.7107 Å). The international tables of X-ray crystallography were consulted for the atoms and anomalous dispersion corrections.^77^ Reduction and data integration were done on SAINT software.^78^ The collected reflections were subjected to empirical absorption using SADABS, and the space group was identified using XPREP.^79^ Furthermore, Olex2 software with the Olex2 solve structure solution program using charge flipping was used to solve the X-ray crystal structure of **L1** and complexes **1** and **2**. The data were refined with the Olex2 refine refinement package using Gauss–Newton (G–N) minimization.^80^ All of the non-hydrogen atoms of synthesized complexes were refined anisotropically. A detailed summary of selected crystallographic parameters is described in Table 1.

**Table 1:** X-ray Crystallographic Details and Single-Crystal Structure Refinement Parameters of Complexes **1** and **2**

parameters	complex **1**	complex **2**
CCDC no.	2191274	2191275
empirical formula	C_60_H_56_Mn_2_N_16_O_12_	C_30_H_22_N_8_O_2_Zn
formula weight	1303.08	591.92
temperature, K	293	293
crystal system	monoclinic	monoclinic
space group	*I*2/*a*	*P*21/*n*
*a*, Å	15.0287 (7)	12.4274(10)
*b*, Å	24.1629(8)	13.3512(5)
*c*, Å	16.1272(9)	12.4653(9)
α, deg	90	90
β, deg	90.127(4)	118.981(10)
γ, deg	90	90
*Z*	4	4
volume, Å^3^	5856.4(5)	1809.3(3)
ρ_calc_, g/cm^3^	1.451	1.087
μ, mm^–1^	0.510	0.712
*F*(000)	2696.0	608.0
crystal size, mm^3^	0.30 × 0.16 × 0.20	0.21 × 0.18 × 0.15
radiation	Mo Kα (0.71073)	Mo Kα (0.71073)
2Θ range for data collection, deg	3.04–62.28	3.798–54.332
index ranges	–15 ≤ *h* ≤ 21, –31 ≤ *k* ≤ 35, –22 ≤ *l* ≤ 13	–15 ≤ *h* ≤ 15, –16 ≤ *k* ≤ 16, –15 ≤ *l* ≤ 15
reflections collected	26 812	26 455
independent reflections	8494 [*R*_int_ = 0.0773, *R*_sigma_ = 0.0951]	3825 [*R*_int_ = 0.0675, *R*_sigma_ = 0.0696]
data/restrains/parameters	8494/0/505	3825/0/187
goodness-of-fit on *F*^2^^a^	1.163	1.049
final *R* indexes [*I* > = 2σ(*I*)]	*R*_1_ = 0.1119, w*R*_2_ = 0.3077	*R*_1_ = 0.1119, w*R*_2_ = 0.2142
final *R* indexes (all data)^b^	*R*_1_ = 0.1831, w*R*_2_ = 0.3790	*R*_1_ = 0.1144, w*R*_2_ = 0.2412
large diff. peak/hole, E å^–3^	5.34/–1.30	0.64/–0.33

a GoF = {∑[*w*(*F*_o_^2^ – *F*_c_^2^)]/(*n* – *p*)}^1/2^, where *p* and *n* denote the number of parameters and number of data, respectively.

b *R* = {∑||*F*_o_| – |*F*_c_||/∑|*F*_o_|}, w*R*_2_ = {∑*w*(*F*_o_^2^ – *F*_c_^2^)2/∑*w*(*F*_o_^2^)_2_}^1/2^.

### 9.5 DFT Studies

The ORCA 3.0.1 software package was employed for the DFT calculations of **L1** and complexes **1** and **2**.^81^ The CIF files of synthesized compounds were directly utilized to obtain optimized geometries by utilizing the B3LYP hybrid functional method. The optimized structures of **L1** and complexes **1** and **2** were used to obtain the HOMO and LUMO electron density with their corresponding energies by employing Aldrich’s def-2TZVP and def2-SVP basis sets to perform the single point energy calculations. The resolution of identity (RI) was employed in conjunction with the auxiliary def2TZV/J or def2-SVP/J Coulomb fitting basis sets to speed up calculations.^82^ Avagadro software version 4.1 was used to generate HOMO–LUMO contour plots of various molecular orbitals. The following equations were used to calculate the various thermodynamic parameters from the respective HOMO–LUMO gaps of synthesized compounds.^83^
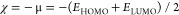
1
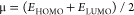
2
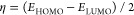
3
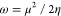
4

### 9.6 Hirshfeld Surface Analysis

The Crystal Explorer 17. 5.27 software program was utilized to examine the intermolecular interactions in the crystal lattice. The s-XRD files of **L1** and complexes **1** and **2** were directly used as the input file in this calculation.^38^ The properties of the Hirshfeld surface include di, de, dnorm, curvedness, and shape index. di denotes the distance from the Hirshfeld surface to the closest atom internal to the surface, de denotes the distance from the Hirshfeld surface to the adjoining atom external to the surface, and dnorm is the normalized sum of di and de. Curvedness is a function dependent on Hirshfeld surface concavity or convexity, and shape index is the function dependent on the flatness or curvature of the Hirshfeld surface.

## 10 *In Vitro* DNA and Protein Binding Studies

All of the binding analyses for **L1** and complexes **1** and **2** were performed at RT in Tris-HCl buffer (pH 7.3) and conformed to the standard procedures used in our lab previously.^84^

To quantify the binding strength of synthesized compounds, the Wolfe–Shimmer equation was used.^46^
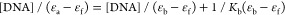
5where ε_a_, ε_b_, and ε_f_ represent the apparent (*A*_abs_/[complex]), bound, and free complex extinction coefficients, respectively, and [DNA] represents the concentration of ct-DNA. The intrinsic binding constant (*K*_b_) value of the synthesized compounds was evaluated from the ratio of the slope of 1/(ε_b_ – ε_f_) to the intercept 1/*K*_b_(ε_b_ – ε_f_) in a plot of [DNA]/(ε_a_ – ε_f_) vs [DNA].

Moreover, in the competitive binding studies (EB studies) performed using emission spectroscopy, the magnitude of binding affinity was calculated by employing the Stern–Volmer equation as described below.^49^
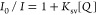
6where *I*_0_ and *I* are the intensities in the absence and presence of EB, respectively, [*Q*] is the concentration of quencher, and *K*_sv_ is the Stern–Volmer binding constant.

The protein (BSA) concentration was measured after the stock solution was prepared in Tris-HCl buffer (pH 7.3) at RT using the absorption coefficient of 35 219 M^–1^ cm^–1^ 280 nm.^58^ Using the following equations and considering that BSA and metal complexes have only one type of binding interaction, the value of *K*_b_ was quantitatively determined.complex + BSA = complex:BSA
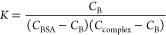
7where *C*_BSA_ is the concentration of BSA, *C*_complex_ is the analytical concentration of synthesized compounds, *K* is the binding constant, and *C*_B_ represents the amount of [complex:BSA] present in the solution. According to the Beer–Lambert law
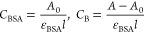
where ε_BSA_ is the molar extinction coefficient of BSA, *l* is the path length of the cuvette in cm, and *A* and *A*_0_ are the absorbances of BSA in the presence and absence of a complex at 280 nm, respectively.

Now, putting the value of *C*_B_ and *C*_BSA_ in eq 7, we get the following equation
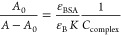
8From eq 8, the double reciprocal plot of 1/*A* – *A*_0_ vs 1/*C*_complex_ is linear, and *K*_b_ can be calculated from the ratio of the slope to that of the intercept.

The molecular docking studies of **L1** and complexes **1** and **2** were performed by employing AutoDock Vina and AutoDock tools 1.5.6.^85^ From the protein data bank (https://www.rcsb.org/pdb), the structure of the receptor molecules B-DNA (PDB ID: 1BNA) and BSA (PDB ID: 4F5S) was collected. During docking studies, when 100% output was done, Autodock vina produced nine types of binding conformations with different binding energies, and the docked model having the lowest energy was chosen for studies. The docked poses of **L1** and complexes **1** and **2** were visualized by the Discovery studio 3.5 molecular graphic program.

## 11 Antioxidant Activity

The 2,2-diphenyl-picrylhydrazyl (DPPH) free radical scavenging activity of **L1** and complexes **1** and **2** was evaluated using spectrophotometry.^86^ Different concentrations (5–35 μM) of ascorbic acid (as standard), **L1**, and complexes **1** and **2** were used for the study. This solution was then added with a DPPH solution (0.2 mM in ethanol), and then, the resulting solution was incubated for 30 min at RT in the dark. At 517 nm, the absorbance of the various solutions and the control (DPPH) was measured, and eq 9 was used to quantify the percent inhibition of the DPPH radical scavenger.
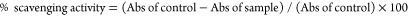
9

## 12 Antibacterial Activity

The antibacterial activities of **L1** and complexes **1** and **2** were assessed by the agar well diffusion method.^87^**L1** and complexes **1** and **2** were dissolved in DMSO, and the stock solution was prepared. DMSO solvent was used as a control. The antibacterial activity of compounds was confirmed by calculating the MIC values using the broth dilution method containing the concentrations of 2, 5, 10, and 15 mM in DMSO.^88^ These measurements were carried out in triplicate for each compound, and their average values are reported.

## 13 *In Vitro* Cytotoxicity Assessment

In this current study, we have used MDA-MB-231 and A549 cell lines derived from breast cancer and lung cancer cells for screening the anticancer activity of synthesized compounds. MDA-MB-231 and A549 cells were grown into 96-well tissue culture plates at a density of 103 cells per well in a growth medium containing 5% serum. Incubation of cancer cells was done overnight at 37 °C in a CO_2_ incubator. The next step was to treat the adhered cells for 48 h with a vehicle containing DMSO and various substances at concentrations ranging up to 20 μM. After 48 h of treatment, the tetrazolium salt 3[4,5-diethylthiazol-2-yl]-2,5-diphenyltetrazolium bromide (MTT) assay was used to evaluate the viability of the cells. Optical densities of all of the samples were evaluated at 450 nm spectrophotometrically, and the results were evaluated in terms percentage of cell viability in comparison to the control. Prism software version 5.0 was used to analyze the data (Graph Pad software, San Diego, CA). All results were presented as mean ± SEM. Following a one-way analysis of variance (ANOVA), Turkey’s post hoc test for multiple comparisons was used to establish the significance of the difference. Statistical significance was defined as a value of *p* < 0.05.^89^

## Supporting Information Available

FTIR, ^1^H and ^13^C NMR, UV–vis spectra, EPR spectra of complex **1**, DFT data, crystallographic details of **L1**, selected bond lengths and bond angles of **L1** and complexes **1** and **2**, emission spectra of complexes **1** and **2**, antioxidant data, cytotoxicity data (PDF)

## References

[ref1] Mjos K. D.; Orvig C. Metallodrugs in medicinal inorganic chemistry. Chem. Rev. 2014, 114, 4540–4563. 10.1021/cr400460s. 24456146

[ref2] Fuertes M. A.; Alonso C.; Perez J. M. Biochemical modulation of cisplatin mechanisms of action: enhancement of antitumor activity and circumvention of drug resistance. Chem. Rev. 2003, 103, 645–662. 10.1021/cr020010d. 12630848

[ref3] aSantini C.; Pellei M.; Gandin V.; Porchia M.; Tisato F.; Marzano C. Advances in copper complexes as anticancer agents. Chem. Rev. 2014, 114, 815–862. 10.1021/cr400135x. 24102434

[ref4] Zhong X.; Wei H. L.; Liu W. S.; Wang D. Q.; Wang X. The crystal structures of copper (II), manganese (II), and nickel (II) complexes of a (Z)-2-hydroxy-N′-(2- oxoindolin-3-ylidene) benzohydrazide—potential antitumor agents. Bioorg. Med. Chem. Lett. 2007, 17, 3774–3777. 10.1016/j.bmcl.2007.04.006. 17466518

[ref5] aHaghdoost M. M.; Guard J.; Golbaghi G.; Castonguay A. Anticancer activity and catalytic potential of ruthenium (II)–arene complexes with N, O-donor ligands. Inorg. Chem. 2018, 57, 7558–7567. 10.1021/acs.inorgchem.8b00346. 29888595

[ref6] aKhare E.; Holten-Andersen N.; Buehler M. J. Transition-metal coordinate bonds for bioinspired macromolecules with tunable mechanical properties. Nat. Rev. Mater. 2021, 6, 421–436. 10.1038/s41578-020-00270-z.

[ref7] aOrvig C.; Abrams M. J. Medicinal inorganic chemistry: introduction. Chem. Rev. 1999, 99, 2201–2204. 10.1021/cr980419w. 11749478

[ref8] aKropp H.; King A. E.; Khusniyarov M. M.; Heinemann F. W.; Lancaster K. M.; DeBeer S.; Bill E.; Meyer K. Manganese nitride complexes in oxidation states III, IV, and V: synthesis and electronic structure. J. Am. Chem. Soc. 2012, 134, 15538–15544. 10.1021/ja306647c. 22920682

[ref9] aIranzo O. Manganese complexes displaying superoxide dismutase activity: a balance between different factors. Bioorg. Chem. 2011, 39, 73–87. 10.1016/j.bioorg.2011.02.001. 21397291

[ref10] Qin Q. P.; Meng T.; Wei Z. Z.; Zhang C. H.; Liu Y. C.; Liang H.; Chen Z. F. Synthesis, Crystal Structure, Cytotoxicity, and Mechanism of Action of Zn(II), Mn(II), and Fe(III) Complexes with 6-Hydroxyloxoisoaporphine. Eur. J. Inorg. Chem. 2017, 2017, 1824–1834. 10.1002/ejic.201601030.

[ref11] aAschner M.; Guilarte T. R.; Schneider J. S.; Zheng W. Manganese: recent advances in understanding its transport and neurotoxicity. Toxicol. Appl. Pharmacol. 2007, 221, 131–147. 10.1016/j.taap.2007.03.001. 17466353 PMC1950780

[ref12] Yao L.; Chen Q. Y.; Xu X. L.; Li Z.; Wang X. M. Interaction of manganese(II) complex with apotransferrin and the apotransferrin enhanced anticancer activities. Spectrochim. Acta, Part A 2013, 105, 207–212. 10.1016/j.saa.2012.12.029. 23314213

[ref13] aMilbeo P.; Quintin F.; Moulat L.; Didierjean C.; Martinez J.; Bantreil X.; Calmès M.; Lamaty F. Synthesis, characterisation and cytotoxic activity evaluation of new metal- salen complexes based on the 1, 2-bicyclo [2.2.2] octane bridge. Tetrahedron Lett. 2021, 63, 152706–152710. 10.1016/j.tetlet.2020.152706.

[ref14] Ansari K. I.; Kasiri S.; Grant J. D.; Mandal S. S. Apoptosis and anti-tumour activities of manganese (III)-salen and-salphen complexes. Dalton Trans. 2009, 40, 8525–8531. 10.1039/b905276c. 19809727

[ref15] aMcCall K. A.; Huang C. C.; Fierke C. A. Zinc and health: current status and future directions. J. Nutr. 2000, 130, 1437–1446. 10.1093/jn/130.5.1437S. 10801957

[ref16] ad’Angelo J.; Morgant G.; Ghermani N. E.; Desmaele D.; Fraisse B.; Bonhomme F.; Dichi E.; Sghaier M.; Li Y.; Journaux Y.; Sorenson J. R. J. Crystal structures and physico-chemical properties of Zn(II) and Co(II) tetraaqua(3-nitro-4- hydroxybenzoato) complexes: their anticonvulsant activities as well as related (5- nitrosalicylato)–metal complexes. Polyhedron 2008, 27, 537–546. 10.1016/j.poly.2007.10.006.

[ref17] Kasuga N. C.; Sekino K.; Ishikawa M.; Honda A.; Yokoyama M.; Nakano S.; Shimada N.; Koumo C.; Nomiya K. Synthesis, structural characterization and antimicrobial activities of 12 zinc (II) complexes with four thiosemicarbazone and two semicarbazone ligands. J. Inorg. Biochem. 2003, 96, 298–310. 10.1016/S0162-0134(03)00156-9. 12888265

[ref18] Pellei M.; Del Bello F.; Porchia M.; Santini C. Zinc coordination complexes as anticancer agents. Coord. Chem. Rev. 2021, 445, 214088 10.1016/j.ccr.2021.214088.

[ref19] aHassan A. M.; Said A. O.; Heakal B. H.; Younis A.; Aboulthana W. M.; Mady M. F. Green Synthesis, Characterization, Antimicrobial and Anticancer Screening of New Metal Complexes Incorporating Schiff Base. ACS Omega 2022, 7, 32418–32431. 10.1021/acsomega.2c03911. 36120022 PMC9475620

[ref20] Kumari P.; Ansari S. N.; Kumar R.; Saini A. K.; Mobin S. M. Design and construction of Aroyl-Hydrazone Derivatives: Synthesis, Crystal Structure, Molecular Docking and Their Biological Activities. Chem. Biodivers. 2019, 16, 1900315 10.1002/cbdv.201900315. 31532059

[ref21] aAvaji P. G.; Kumar C. V.; Patil S. A.; Shivananda K. N.; Nagaraju C. Synthesis, spectral characterization, *in-vitro* microbiological evaluation and cytotoxic activities of novel macrocyclic bis hydrazone. Eur. J. Med. Chem. 2009, 44, 3552–3559. 10.1016/j.ejmech.2009.03.032. 19419802

[ref22] aKüçükgüzel Ş.; Oruc E. E.; Rollas S.; Sahin F.; Ozbek A. Synthesis, characterisation and biological activity of novel 4-thiazolidinones, 1, 3, 4-oxadiazoles and some related compounds. Eur. J. Med. Chem. 2002, 37, 197–206. 10.1016/S0223-5234(01)01326-5. 11900864

[ref23] aXia L. Y.; Wang W. L.; Wang S. H.; Huang Y. L.; Shan S. N′-[(E)-3-Indol-3- ylmethylene] isonicotinohydrazide monohydrate. Acta Crystallogr., Sect. E 2009, E65, o1900 10.1107/S1600536809027329. PMC2977231 21583590

[ref24] aQiu-Yun C.; Dong-Fang Z.; Juan H.; Wen-Jie G.; Jing G. Synthesis, anticancer activities, interaction with DNA and mitochondria of manganese complexes. J. Inorg. Biochem. 2010, 104, 1141–1147. 10.1016/j.jinorgbio.2010.06.012. 20674030

[ref25] El-Shwiniy W. H.; Shehab W. S.; Zordok W. A. Spectral, thermal, DFT calculations,anticancer and antimicrobial studies for bivalent manganese complexes of pyrano [2, 3-d] pyrimidine derivatives. J. Mol. Struct. 2020, 1199, 126993–127007. 10.1016/j.molstruc.2019.126993.

[ref26] Pouralimardan O.; Chamayou A. C.; Janiak C.; Hosseini-Monfared H. Hydrazone Schiff base-manganese (II) complexes: Synthesis, crystal structure and catalytic reactivity. Inorg. Chim. Acta 2007, 360, 1599–1608. 10.1016/j.ica.2006.08.056.

[ref27] Fekri R.; Salehi M.; Asadi A.; Kubicki M. Synthesis, characterization, anticancer and antibacterial evaluation of Schiff base ligands derived from hydrazone and their transition metal complexes. Inorg. Chim. Acta 2019, 484, 245–254. 10.1016/j.ica.2018.09.022.

[ref28] Stevanović N.; Zlatar M.; Novaković I.; Pevec A.; Radanović D.; Matić I. Z.; Đorđić Crnogorac M.; Crnogorac M. D.; Stanojkovic T.; Vujcic M.; Gruden M.; Sladic D.; Anđelković K.; Cobeljic B. Cu (II), Mn (II) and Zn (II) complexes of hydrazones with a quaternary ammonium moiety: synthesis, experimental and theoretical characterization and cytotoxic activity. Dalton Trans. 2021, 51, 185–196. 10.1039/D1DT03169D. 34877947

[ref29] Dasgupta S.; Karim S.; Banerjee S.; Saha M.; Saha K. D.; Das D. Designing of novel zinc (II) Schiff base complexes having acyl hydrazone linkage: study of phosphatase and anti-cancer activities. Dalton Trans. 2020, 49, 1232–1240. 10.1039/C9DT04636D. 31903474

[ref30] aEscriche-Tur L.; Font-Bardia M.; Albela B.; Corbella M. Determination of ZFS parameters from the EPR spectra of mono-, di-and trinuclear Mn (II) complexes: impact of magnetic coupling. Dalton Trans. 2017, 46, 2699–2714. 10.1039/C6DT04012H. 28170010

[ref31] aKose M.; Goring P.; Lucas P.; Mckee V. Mono-, di-and tri-nuclear manganese (II) complexes derived from a quinquedentate ligand: Superoxide dismutase and catalase mimetic studies. Inorg. Chim. Acta 2015, 435, 232–238. 10.1016/j.ica.2015.07.010.

[ref32] Bhar K.; Sutradhar D.; Choubey S.; Ghosh R.; Lin C. H.; Ribas J.; Ghosh B. K. Hexa and heptacoordinated manganese (II) dicyanamide complexes containing a tetradentate N-donor Schiff base: Syntheses, composition tailored architectures and magnetic properties. J. Mol. Struct. 2013, 1051, 107–114. 10.1016/j.molstruc.2013.07.029.

[ref33] aHujon F.; Lyngdoh R. D.; King R. B. Metal–metal bond distances and bond orders in dimanganese complexes with bidentate ligands: scope for some very short Mn–Mn bonds. New J. Chem. 2020, 44, 12993–13006. 10.1039/D0NJ01305F.

[ref34] aMaiti M.; Sadhukhan D.; Thakurta S.; Zangrando E.; Pilet G.; Bauzá A.; Frontera A.; Dede B.; Mitra S. Synthesis, structural characterization, theoretical calculations and catecholase mimetic activity of manganese-Schiff base complexes. Polyhedron 2014, 75, 40–49. 10.1016/j.poly.2014.03.005.

[ref35] aZianna A.; Psomas G.; Hatzidimitriou A.; Coutouli-Argyropoulou E.; Lalia- Kantouri M. Zinc complexes of salicylaldehydes: synthesis, characterization and DNA- binding properties. J. Inorg. Biochem. 2013, 127, 116–126. 10.1016/j.jinorgbio.2013.07.031. 23973683

[ref36] aStevanović N.; Zlatar M.; Novaković I.; Pevec A.; Radanović D.; Matić I. Z.; Crnogorac M. D.; Stanojković T.; Vujcic M.; Gruden M.; Sladic D.; Anđelkovic; Iztok T. K.; Cobeljic B. (). Cu (II), Mn (II) and Zn (II) complexes of hydrazones with a quaternary ammonium moiety: synthesis, experimental and theoretical characterization and cytotoxic activity. Dalton Trans. 2021, 51, 185–196. 10.1039/D1DT03169D. 34877947

[ref37] Anyama C. A.; Ita B. I.; Ayi A. A.; Louis H.; Okon E. E.; Ogar J. O.; Oseghale C. O. Experimental and density functional theory studies on a zinc (II) coordination polymer constructed with 1, 3, 5-benzenetricarboxylic acid and the derived nanocomposites from activated carbon. ACS Omega 2021, 6, 28967–28982. 10.1021/acsomega.1c04037. 34746588 PMC8567384

[ref38] Cuenú F.; Londono-Salazar J.; Torres J. E.; Abonia R.; D’Vries R. F. Synthesis, structural characterization and theoretical studies of a new Schiff base 4-(((3-(tert- Butyl)- (1-phenyl) pyrazol-5-yl) imino) methyl) phenol. J. Mol. Struct. 2018, 1152, 163–176. 10.1016/j.molstruc.2017.09.078.

[ref39] Yusuf T. L.; Oladipo S. D.; Zamisa S.; Kumalo H. M.; Lawal I. A.; Lawal M. M.; Mabuba N. Design of new Schiff-Base Copper (II) complexes: Synthesis, crystal structures, DFT study, and binding potency toward cytochrome P450 3A_4_. ACS Omega 2021, 6, 13704–13718. 10.1021/acsomega.1c00906. 34095663 PMC8173565

[ref40] Spackman M. A.; Jayatilaka D. Hirshfeld surface analysis. CrystEngComm 2009, 11, 19–32. 10.1039/B818330A.

[ref41] Clausen H. F.; Chevallier M. S.; Spackman M. A.; Iversen B. B. Three new co- crystals of hydroquinone: crystal structures and Hirshfeld surface analysis of intermolecular interactions. New J. Chem. 2010, 34, 193–199. 10.1039/B9NJ00463G.

[ref42] Seth S. K.; Sarkar D.; Kar T. Use of π–π forces to steer the assembly of chromone derivatives into hydrogen bonded supramolecular layers: crystal structures and Hirshfeld surface analyses. CrystEngComm 2011, 13, 4528–4535. 10.1039/c1ce05037k.

[ref43] aLiu H. K.; Sadler P. J. Metal complexes as DNA intercalators. Acc. Chem. Res. 2011, 44, 349–359. 10.1021/ar100140e. 21446672

[ref44] aKumar P.; Gorai S.; Santra M. K.; Mondal B.; Manna D. DNA binding, nuclease activity and cytotoxicity studies of Cu (II) complexes of tridentate ligands. Dalton Trans. 2012, 41, 7573–7581. 10.1039/c2dt30232b. 22588369

[ref45] aGhosh M. K.; Pathak S.; Ghorai T. K. Synthesis of Two Mononuclear Schiff Base Metal (M = Fe, Cu) complexes: MOF Structure, Dye Degradation, H2O2 Sensing, and DNA Binding Property. ACS Omega 2019, 4, 16068–16079. 10.1021/acsomega.9b02268. 31592474 PMC6777120

[ref46] Wolfe A.; Shimer G. H. Jr.; Meehan T. Polycyclic aromatic hydrocarbons physically intercalate into duplex regions of denatured DNA. Biochemistry 1987, 26, 6392–6396. 10.1021/bi00394a013. 3427013

[ref47] aYan Y.; Zhang J.; Ren L.; Tang C. Metal-containing and related polymers for biomedical applications. Chem. Soc. Rev. 2016, 45, 5232–5263. 10.1039/C6CS00026F. 26910408 PMC4996776

[ref48] Healy E. F. Quantitative determination of DNA–ligand binding using fluorescence spectroscopy. J. Chem. Educ. 2007, 84, 1304–1307. 10.1021/ed084p1304.

[ref49] aKoumousi E. S.; Zampakou M.; Raptopoulou C. P.; Psycharis V.; Beavers C. M.; Teat S. J.; Psomas G.; Stamatatos T. C. First palladium(II) and platinum(II) complexes from employment of 2, 6-diacetylpyridine dioxime: synthesis, structural and spectroscopic characterization, and biological evaluation. Inorg. Chem. 2012, 51, 7699–7710. 10.1021/ic300739x. 22742945

[ref50] Dimiza F.; Fountoulaki S.; Papadopoulos A. N.; Kontogiorgis C. A.; Tangoulis V.; Raptopoulou C. P.; Psycharis V.; Terzis A.; Kessissoglou D. P.; Psomas G. Non- steroidal antiinflammatory drug–copper (II) complexes: structure and biological perspectives. Dalton Trans. 2011, 40, 8555–8568. 10.1039/c1dt10714c. 21805007

[ref51] Zhao D.; Wu Y.; Huang W.; Gong S.; Chen Z. DNA binding, DNA cleavage, cellular uptake, cytotoxicity, and apoptosis-inducing ability of a binuclear Schiff base copper(II) complex. New J. Chem. 2022, 46, 15219–15226. 10.1039/D2NJ03077B.

[ref52] aZheng K.; Liu F.; Xu X. M.; Li Y. T.; Wu Z. Y.; Yan C. W. Synthesis, structure and molecular docking studies of dicopper(II) complexes bridged by N- phenolato-N′-[2-(dimethylamino) ethyl] oxamide: the influence of terminal ligands on cytotoxicity and reactivity towards DNA and protein BSA. New J. Chem. 2014, 38, 2964–2978. 10.1039/C4NJ00092G.

[ref53] aZampakou M.; Akrivou M.; Andreadou E. G.; Raptopoulou C. P.; Psycharis V.; Pantazaki A. A.; Psomas G. Structure, antimicrobial activity, DNA-and albumin- binding of manganese (II) complexes with the quinolone antimicrobial agents oxolinic acid and enrofloxacin. J. Inorg. Biochem. 2013, 121, 88–99. 10.1016/j.jinorgbio.2012.12.013. 23353085

[ref54] aLi Y.; Yang Z.; Zhou M.; He J.; Wang X.; Wu Y.; Wang Z. Syntheses, crystal structures and DNA-binding studies of Cu (II) and Zn (II) complexes bearing asymmetrical aroylhydrazone ligand. J. Mol. Struct. 2017, 1130, 818–828. 10.1016/j.molstruc.2016.10.092.

[ref55] Loganathan R.; Ramakrishnan S.; Suresh E.; Palaniandavar M.; Riyasdeen A.; Akbarsha M. A. Mixed ligand μ-phenoxo-bridged dinuclear copper (II) complexes with diimine co-ligands: efficient chemical nuclease and protease activities and cytotoxicity. Dalton Trans. 2014, 43, 6177–6194. 10.1039/c3dt52518j. 24595529

[ref56] aUma Maheswari P.; Palaniandavar M. DNA binding and cleavage properties of certain tetrammine ruthenium (II) complexes of modified 1, 10-phenanthrolines–effect of hydrogen-bonding on DNA-binding affinity. J. Inorg. Biochem. 2004, 98, 219–230. 10.1016/j.jinorgbio.2003.09.003. 14729302

[ref57] Iqbal H.; Yang T.; Li T.; Zhang M.; Ke H.; Ding D.; Deng Y.; Chen H. Serum protein-based nanoparticles for cancer diagnosis and treatment. J. Controlled Release 2021, 329, 997–1022. 10.1016/j.jconrel.2020.10.030. 33091526

[ref58] Carter D. C.; Ho J. X. Structure of serum albumin. Adv. Protein Chem. 1994, 45, 153–203. 10.1016/S0065-3233(08)60640-3. 8154369

[ref59] aChudzik M.; Maciązek-Jurczyk M.; Pawełczak B.; Sułkowska A. Spectroscopic studies on the molecular ageing of serum albumin. Molecules 2017, 22, 34–48. 10.3390/molecules22010034. PMC6155906 28035999

[ref60] aGreenfield N. J. Using circular dichroism spectra to estimate protein secondary structure. Nat. Protoc. 2006, 1, 2876–2890. 10.1038/nprot.2006.202. 17406547 PMC2728378

[ref61] Xiang Y.; Wu F. Study of the interaction between a new Schiff-base complex and bovine serum albumin by fluorescence spectroscopy. Spectrochim. Acta, Part A 2010, 77, 430–436. 10.1016/j.saa.2010.06.010. 20598629

[ref62] Sahoo B. K.; Ghosh K. S.; Bera R.; Dasgupta S. Studies on the interaction of diacetylcurcumin with calf thymus-DNA. Chem. Phys. 2008, 351, 163–169. 10.1016/j.chemphys.2008.05.008.

[ref63] Dhanaraj C. J.; Johnson J. Quinoxaline based bio-active mixed ligand transition metal complexes: Synthesis, characterization, electrochemical, antimicrobial, DNA binding, cleavage, antioxidant and molecular docking studies. J. Photochem. Photobiol. B 2015, 151, 100–109. 10.1016/j.jphotobiol.2015.07.010. 26232747

[ref64] de Torre M. P.; Cavero R. Y.; Calvo M. I.; Vizmanos J. L. A simple and a reliable method to quantify antioxidant activity *in vivo*. Antioxidants 2019, 8, 142 10.3390/antiox8050142. 31121854 PMC6562907

[ref65] Hasi Q. M.; Fan Y.; Yao X. Q.; Hu D. C.; Liu J. C. Synthesis, characterization, antioxidant and antimicrobial activities of a bidentate Schiff base ligand and its metal complexes. Polyhedron 2016, 109, 75–80. 10.1016/j.poly.2016.01.052.

[ref66] Li Y.; Yang Z. Y.; Wu J. C. Synthesis, crystal structures, biological activities and fluorescence studies of transition metal complexes with 3-carbaldehyde chromone thiosemicarbazone. Eur. J. Med. Chem. 2010, 45, 5692–5701. 10.1016/j.ejmech.2010.09.025. 20884087

[ref67] Colinas I. R.; Rojas-Andrade M. D.; Chakraborty I.; Oliver S. R. Two structurally diverse Zn-based coordination polymers with excellent antibacterial activity. CrystEngComm 2018, 20, 3353–3362. 10.1039/C8CE00394G.

[ref68] aAnacona J. R.; Rodriguez J. L.; Camus J. Synthesis, characterization and antibacterial activity of a Schiff base derived from cephalexin and sulphathiazole and its transition metal complexes. Spectrochim. Acta, Part A 2014, 129, 96–102. 10.1016/j.saa.2014.03.019. 24727167

[ref69] Zampakou M.; Akrivou M.; Andreadou E. G.; Raptopoulou C. P.; Psycharis V.; Pantazaki A. A.; Psomas G. Structure, antimicrobial activity, DNA and albumin- binding of manganese (II) complexes with the quinolone antimicrobial agents oxolinic acid and enrofloxacin. J. Inorg. Biochem. 2013, 121, 88–99. 10.1016/j.jinorgbio.2012.12.013. 23353085

[ref70] Efthimiadou E. K.; Karaliota A.; Psomas G. Mononuclear metal complexes of the second-generation quinolone antibacterial agent enrofloxacin: Synthesis, structure, antibacterial activity and interaction with DNA. Polyhedron 2008, 27, 1729–1738. 10.1016/j.poly.2008.02.006.

[ref71] Nitha L. P.; Aswathy R.; Mathews N. E.; Sindhu Kumari B.; Mohanan K. Synthesis, spectroscopic characterisation, DNA cleavage, superoxidase dismutase activity and antibacterial properties of some transition metal complexes of a novel bidentate Schiff base derived from isatin and 2-aminopyrimidine. Spectrochim. Acta, Part A 2014, 118, 154–161. 10.1016/j.saa.2013.08.075. 24051284

[ref72] Murugaiyan M.; Mani S. P.; Sithique M. A. Zinc (II) centered biologically active novel N, N, O donor tridentate water-soluble hydrazide-based O-carboxymethyl chitosan Schiff base metal complexes: synthesis and characterisation. New J. Chem. 2019, 43, 9540–9554. 10.1039/C9NJ00670B. 31220500

[ref73] Liu W.; Bensdorf K.; Proetto M.; Abram U.; Hagenbach A.; Gust R. NHC gold halide complexes derived from 4, 5-diarylimidazoles: synthesis, structural analysis, and pharmacological investigations as potential antitumor agents. J. Med. Chem. 2011, 54, 8605–8615. 10.1021/jm201156x. 22091836

[ref74] aNiu M.; Hong M.; Chang G.; Li X.; Li Z. A comparative study of cytotoxicity and interaction with DNA/protein of five transition metal complexes with Schiff base ligands. J. Photochem. Photobiol. B 2015, 148, 232–241. 10.1016/j.jphotobiol.2015.04.023. 25974907

[ref75] Dasgupta S.; Karim S.; Banerjee S.; Saha M.; Saha K. D.; Das D. Designing of novel zinc (II) Schiff base complexes having acyl hydrazone linkage: study of phosphatase and anti-cancer activities. Dalton Trans. 2020, 49, 1232–1240. 10.1039/C9DT04636D. 31903474

[ref76] Baharuddin P.; Satar N.; Fakiruddin K. S.; Zakaria N.; Lim M. N.; Yusoff N. M.; Zakaria Z.; Yahaya B. H. Curcumin improves the efficacy of cisplatin by targeting cancer stem-like cells through p21 and cyclin D1-mediated tumour cell inhibition in non-small cell lung cancer cell lines. Oncol. Rep. 2016, 35, 13–25. 10.3892/or.2015.4371. 26531053 PMC4699625

[ref77] Milburn G. International Tables for X-Ray Crystallography, Struct. Sci.; 1983.

[ref78] Sheldrick G. M. SADABS: Empirical Absorption Correction Program; University of Göttingen: Göttingen, Germany, 1997.

[ref79] aXPREP, version 5.1; Siemens Industrial Automation Inc.: Madison, WI, 1995.

[ref80] aDolomanov O. V.; Bourhis L. J.; Gildea R. J.; Howard J. A.; Puschmann H. OLEX2: a complete structure solution, refinement and analysis program. J. Appl. Crystallogr. 2009, 42, 339–341. 10.1107/S0021889808042726.

[ref81] aComba P.; Hausberg S.; Martin B. Calculation of exchange coupling constants of transition metal complexes with DFT. J. Phys. Chem. A 2009, 113, 6751–6755. 10.1021/jp900752p. 19469514

[ref82] aNeese F.; Wennmohs F.; Becker U.; Riplinger C. The ORCA quantum chemistry program package. J. Chem. Phys. 2020, 152, 224108 10.1063/5.0004608. 32534543

[ref83] aAdejumo T. T.; Tzouras N. V.; Zorba L. P.; Radanovic D.; Pevec A.; Grubisic S.; Mitic D.; Andelkovic K. K.; Vougioukalakis G. C.; Cobeljic B.; Turel I. Synthesis, characterization, catalytic activity, and DFT calculations of Zn(II) hydrazone complexes. Molecules 2020, 25, 4043–4061. 10.3390/molecules25184043. 32899683 PMC7570652

[ref84] aYousuf I.; Usman M.; Ahmad M.; Tabassum S.; Arjmand F. Single X-ray crystal structure, DFT studies and topoisomerase I inhibition activity of a tailored ionic Ag (I) nalidixic acid–piperazinium drug entity specific for pancreatic cancer cells. New J. Chem. 2018, 42, 506–519. 10.1039/C7NJ03602G.

[ref85] aTrott O.; Olson A. J. AutoDock Vina: improving the speed and accuracy of docking with a new scoring function, efficient optimization, and multithreading. J. Comput. Chem. 2010, 31, 455–461. 19499576 10.1002/jcc.21334 PMC3041641

[ref86] aHasi Q. M.; Fan Y.; Yao X. Q.; Hu D. C.; Liu J. C. Synthesis, characterization, antioxidant and antimicrobial activities of a bidentate Schiff base ligand and its metal complexes. Polyhedron 2016, 109, 75–80. 10.1016/j.poly.2016.01.052.

[ref87] Magaldi S.; Mata-Essayag S.; De Capriles C. H.; Pérez C.; Colella M. T.; Olaizola C.; Ontiveros Y. Well diffusion for antifungal susceptibility testing. Int. J. Infect. Dis. 2004, 8, 39–45. 10.1016/j.ijid.2003.03.002. 14690779

[ref88] Stevanović N.; Zlatar M.; Novakovic I.; Pevec A.; Radanovic D.; Matic I. Z.; Crnogorac M. D.; Stanojkovic T.; Vujcic M.; Gruden M.; Sladic D.; Anđelkovic K.; Turel I.; Cobeljic B. Cu(II), Mn(II) and Zn(II) complexes of hydrazones with a quaternary ammonium moiety: synthesis, experimental and theoretical characterization and cytotoxic activity. Dalton Trans. 2021, 51, 185–196. 10.1039/D1DT03169D. 34877947

[ref89] Bhat S. A.; Fatima Z.; Sood A.; Shukla R.; Hanif K. The Protective Effects of AT2R Agonist, CGP42112A, Against Angiotensin II-Induced Oxidative Stress and Inflammatory Response in Astrocytes: Role of AT2R/PP2A/NFκB/ROS Signaling. Neurotox. Res. 2021, 39, 1991–2006. 10.1007/s12640-021-00403-4. 34529240

