# Supplementary material for: Syntheses, Structural Characterization, and Cytotoxicity Assessment of Novel Mn(II) and Zn(II) Complexes of Aroyl-Hydrazone Schiff Base Ligand
Source: ACS Omega. 2023 Jan 11;8(3):3026–42. doi: 10.1021/acsomega.2c05927 (PMC9878661; doi:10.1021/acsomega.2c05927)
Supplement: Supplementary file 1 — ao2c05927_si_001.pdf [file ao2c05927_si_001.pdf]

Supplementary information

Syntheses, structural characterization and cytotoxicity assessment of novel Mn(II) and Zn(II) complexes of aroyl–hydrazone Schiff base ligands

Masrat Bashir[a], Aijaz A. Dar[b], Imtiyaz Yousuf[a]*

*Corresponding author *E–mail address*: imtiyazchem@gmail.com. (I. Yousuf)
[a]*Department of Chemistry, Aligarh Muslim University, Aligarh 202002, Uttar Pradesh, India.*
[b]*Department of Chemistry, University of Kashmir*, *Hazratbal, Srinagar 190 006, J&K, India.*

LIST OF TABLES

Title **Page No.**

Table S1: Crystallographic data and structure refinement details of **L1**…………………...S3

Table S2. DFT calculated geometrical parameters of **L1** ……………………………….....S4

Table S3. DFT calculated geometrical parameters of complex **1**…………………………..S4

Table S4. DFT calculated geometrical parameters of complex **2** ……………………….....S5

Table S5. Energy of various FMOs of **L1**, complex **1** and complex **2** ……………………S5

Table S6. Thermodynamic and reactivity parameters of **L1**, complex **1** and complex **2**… .S6

Table S7. Cathodic and Anodic potiential and current for complexes **1** & **2** ……………. .S6

Table S8. DPPH radical scavanging activity of ligand **L1**, complex **1** and complex **2**…... .S6

Table S9. Antibacterial activity of ligand **L1** and its metal complexes **1** and **2**…………....S7

Table S10: Minimum inhibition concentration values of **L1** and complexes **1** & **2**……….S7

Table S11. Percent cell viability of **L1** and complexes **1** & **2** against A549……………....S7

Table S12. Percent cell viability of **L1** and complexes **1** & **2** against MDA-MB-231…....S8

Table S13: Comparison table of IC_{50} values of complex **2**………………………………...S8

LIST OF FIGURES

Title **Page No.**
Figure S1. Solution stability studies of complexes **1** & **2**……………….............................S9

Figure S2. Comparative UV–vis spectra of ligand (**L1**) and complexes **1** & **2**……………..S9

Figure S3. FTIR spectra of ligand **L1** and complexes **1** & **2**………………………………..S10

Figure S4. ^{1}HNMR spectra of ligand **L1**…………………………………………………S10

Figure S5. ^{13}CNMR spectra of ligand **L1**……………………………………………….S11

Figure S6. ^{1}HNMR spectra of complex **2**……………………………………………….S11

Figure S7. ^{13}CNMR spectra of complex **2**……………………………………………….S11

Figure S8. EPR spectra of complex **1**……………………………………………………...S12

Figure S9. Single XRD structure of ligand **L1**…………………………………………..S12

Figure S10. Single XRD image illustrating dihedral angle between two planes……………S12

Figure S11. FMOs of ligand **L1** generated at B3LYP functional…………………………..S13

Figure S12. FMOs of complex **1** generated at B3LYP functional………………………….S13

Figure S13. FMOs of complex **2** generated at B3LYP functional………………………….S14

Figure S14. 3D Hirshfeld surface mapping of **L1** …………………………………………S14

Figure S15. 3D Hirshfeld surface mapping of complex **2**………………………………..S14

Figure S16. 2D fingerprint plots of **L1** and complexes **1** & **2**………………………………S15

Figure S17. Emission spectra of complexes **1** & **2**…………………………………………S15

Figure S18. Emission spectra of EB–DNA system recorded in Tris-HCl buffer……………S16

Figure S19. Cyclic voltammogram curves of complexes **1** and **2**…………………………..S16

Figure S20. CD spectra of ct-DNA of complexes **1** and **2** …………………………………S16

Figure S21. Emission spectra of BSA of complex **1** and **2** …………………………………S17

Figure S22. CD spectrum of BSA alone in the presence and absence complex **1** and **2**……S17

Figure S23. Molecular docked structure of **L1** with DNA…………………………………S18

Figure S24. Molecular docked structure of complex **2** with DNA…………………………S18

Figure S25. Molecular docked structure of **L1** with BSA…………………………………..S19

Figure S26. Molecular docked structure of complex **1** with BSA…………………………..S19

Figure S27. Antibacterial action of control, **L1** and complexes **1** & **2**……………………S20

References……………………………………………………………………………………S20

TABLES

Table S1: Crystallographic data and structure refinement details of **L1**.

Parameters	L1
Empirical formula	$C_{15}H_{12}N_4O$
CCDC No.	2191272
Formula weight	282.30
Temperature/K	293
Crystal system	monoclinic
Space group	$P2_1/c$
a/Å	7.1579(1)
b/Å	25.1674(3)
c/Å	7.9414(1)
α/°	90
β/°	104.060(1)
γ/°	90
Volume/Å^3	1387.75(3)
Z	4
ρ_{calc}g/cm^3	1.351
μ/mm^{-1}	0.766
F(000)	594.0
Crystal size/mm^3	0.28 × 0.21 × 0.16
Radiation	Cu Kα (λ = 1.54184)
2Θ range for data collection/°	7.02 to 136.06
Index ranges	$-6 \le h \le 8$, $-30 \le k \le 30$, $-9 \le l \le 9$
Reflections collected	11590
Independent reflections	2532 [R_{int} = 0.0376, R_{sigma} = 0.0209]
Data/restraints/parameters	2532/0/194
Goodness-of-fit on F^2	1.039
Final R indexes [I>=2σ (I)]	R_1 = 0.0410, wR_2 = 0.1184
Final R indexes [all data]	R_1 = 0.0434, wR_2 = 0.1206
Largest diff. peak/hole/eÅ^{-3}	0.20/-0.18

[a]GoF is defined as $\{\Sigma[w(F_o^2 - F_c^2)]/(n - P)\}^{1/2}$ where n is the number of data and p is the number of parameters. [b]$R = \{\Sigma||F_o| - |F_c||/\Sigma|F_o|$, $wR^2 = \{\Sigma w(F_o^2 - F_c^2)^2/\Sigma w(F_o^2)_2\}^{1/2}$.

Table S2: DFT calculated geometrical parameters of **L1** in comparison to experimental parameters (sXRD).

**Bond angles (°)**		
**Atom-Atom-Atom**	**Experimental**	**Calculated**
C12-N13-C14	109.02(11)	109.05
C10-N9-N8	114.27(12)	114.30
C1-N8-N9	119.02(12)	119.09
C19-C14-N13	107.83(12)	107.89
C4-C14-N13	129.80(12)	129.83
C4-C14-C19	122.37(13)	122.39
C11-C19-C14	106.48(12)	106.48
C18-C19-C14	118.79(12)	118.79
C18-C19-C11	134.69(12)	134.60
C12-C11-C19	109.02(11)	109.05

**Bond length (Å)**					
**Atom-Atom**	**Experimental values**	**Calculated values**	**Atom-Atom**	**Experimental values**	**Calculated values**
N13-C14	1.3790(18)	1.378	C14-C19	1.4097(18)	1.410
N13-C12	1.3510(19)	1.349	C14-C4	1.3916(19)	1.396
O7-C1	1.2244(18)	1.223	C19-C11	1.4395(19)	1.440
N9-N8	1.3952(16)	1.395	C14-C4	1.3988(19)	1.398
N9-C10	1.2783(19)	1.279	C19-C11	1.4383(19)	1.438
N8-C1	1.3380(19)	1.339	C19-C18	1.4097(18)	1.409

Table S3: DFT calculated geometrical parameters of complex **1** in comparison to experimental parameters (sXRD).

**Bond angles (°)**		
**Atom-Atom-Atom**	**Experimental**	**Calculated**
O2Mn1O1	99.55(15)	99.57
O3Mn1O1	89.45(15)	89.44
O3Mn1O1	168.77(15)	168.75
O3Mn1O2	90.58(16)	90.60
O3Mn1O2	169.83(14)	169.83
N3Mn1O1	78.76(15)	78.78
N3Mn1O2	90.90(15)	90.96
N3Mn1O3	96.36(15)	96.39
N3Mn1O3	95.51(15)	95.54
N7Mn1O1	90.91(15)	90.93
N7Mn1O2	78.39(16)	78.38
N7Mn1O3	95.91(15)	95.97
N7Mn1O3	96.88(16)	96.79
N7Mn1N3	163.78(19)	163.79

**Bond distances (Å)**					
**Atom-Atom**	**Experimental values**	**Calculated values**	**Atom-Atom**	**Experimental values**	**Calculated values**
Mn1-O1	1.952(3)	1.953	O1-C6	1.308(5)	1.305
Mn1-O3	1.969(4)	1.958	O2-C21	1.306(5)	1.297
Mn1-O3	1.831(4)	1.836	N1-C1	1.333(11)	1.332
Mn1-N3	1.820(3)	1.822	N1-C5	1.321(9)	1.329
Mn1-N7	1.987(4)	1.984	N2-N3	1.393(6)	1.395

Table S4: DFT calculated geometrical parameters of complex **2** in comparison to experimental parameters (sXRD).

**Bond angles (°)**		
**Atom-Atom-Atom**	**Experimental**	**Calculated**
O1-Zn1-O1	180.0	180.00
N1-Zn1-O1	101.23(17)	101.28
N1-Zn1-O1	78.77(17)	78.79
N1-Zn1-N1	180.0	180.0
N1-Zn1-O1	88.36(17)	88.36
N2-Zn1-O1	91.64(17)	91.64
N2-Zn1-O1	91.64(17)	91.66
N1-Zn1-O1	88.36(17)	88.38

**Bond distances (Å)**					
**Atom-Atom**	**Experimental values**	**Calculated values**	**Atom-Atom**	**Experimental values**	**Calculated values**
Zn1-O1	2.060(4)	2.062	N2-N5	1.401(11)	1.401
Zn1-O1	2.060(4)	2.120	N2-C4	1.03(4)	1.032
Zn1-N1	2.069(5)	2.072	N2-C3	1.24(4)	1.233
Zn1-N1	2.069(5)	2.069	O1-C18	1.268(6)	1.261
Zn1-N2	2.343(5)	2.340	N1-N2	1.410(6)	1.414
Zn1-N2	2.343(5)	2.344	N1-C15	1.269(8)	1.272

Table S5: Energy of various FMOs of **L1**, complex **1** and complex **2** and their respective HOMO-LUMO energy gaps.

		**L1**		
**FMO's**	**Energy (eV)**	**FMO's**	**Energy (eV)**	**ΔE**
HOMO	–3.679	LUMO	–1.563	2.116
HOMO-1	–3.373	LUMO+1	–0.854	2.519
HOMO-2	–3.701	LUMO+2	–0.522	3.179
HOMO-3	–3.922	LUMO+3	–0.196	3.726
HOMO-4	–4.038	LUMO+4	–0.548	3.490
		**Complex 1**		
HOMO	–4.243	LUMO	–2.805	1.438
HOMO-1	–4.386	LUMO+1	–2.627	1.759
HOMO-2	–4.585	LUMO+2	–2.061	2.524
HOMO-3	–4.890	LUMO+3	–1.974	2.916
HOMO-4	–5.217	LUMO+4	–1.587	3.630
		**Complex 2**		
HOMO	–3.929	LUMO	–2.677	1.252
HOMO–1	–3.994	LUMO+1	–2.630	1.364
HOMO–2	–4.147	LUMO+2	–2.100	2.047
HOMO 3	–4.569	LUMO+3	–1.733	2.836
HOMO 4	–4.742	LUMO+4	–1.698	3.044

Table S6: Thermodynamic and reactivity parameters of **L1** and complexes **1** & **2** by using B3LYP hybrid functional.

Parameters	L1	Complex 1	Complex 2
LUMO energy (eV)	-1.563	-2.805	-2.677
HOMO energy (eV)	-3.679	-4.243	-3.929
LUMO–HOMO	2.116	1.438	1.252
χ(eV)	2.621	3.524	3.303
μ(eV)	-2.621	-3.524	-3.303
η(eV)	1.058	0.719	0.626
ω(eV)	3.247	8.636	8.714

Table S7: Electrode (cathode and anode) potiential (mV) and current (A) for the redox couples of complexes **1** & **2** with ct-DNA in 5mM Tris-buffer solution (pH 7.3) at a scan rate of 100mV s^{-1}

Complexes	Complex Alone		Complex + ct-DNA		ΔE_{pa}	ΔE_{pc}
	Potential (mV)	Current (A)	Potential (mV)	Current (A)		
1	E_{pa} = 596<br>E_{pc} = 620<br>$E_{1/2}$ = 608<br>ΔE_p = –24	I_{pa} = 1.036 × 10^{-5}<br>I_{pc} = -1.370 × 10^{-5}<br>I_{pa}/I_{pc} = 0.756	E_{pa} = 535<br>E_{pc} = 582<br>$E_{1/2}$ = 559<br>ΔE_p = –47	I_{pa} = 1.838 × 10^{-5}<br>I_{pc} = -1.556 × 10^{-5}<br>I_{pa}/I_{pc} = 1.18	–61	–38
2	E_{pa} = 650<br>E_{pc} = 614<br>$E_{1/2}$ = 632<br>ΔE_p = –36	I_{pa} = 0.906 × 10^{-5}<br>I_{pc} = -1.094 × 10^{-5}<br>I_{pa}/I_{pc} = 0.828	E_{pa} = 618<br>E_{pc} = 603<br>$E_{1/2}$ = 611<br>ΔE_p = –15	I_{pa} = 1.007 × 10^{-5}<br>I_{pc} = -1.352 × 10^{-5}<br>I_{pa}/I_{pc} = 0.744	–32	–11

Table S8: DPPH radical scavanging activity of ligand **L1** and complexes **1** & **2** along with standard ascorbic acid (AA) at different concentrations (λ_{max} = 517 nm). Data represent mean ± SEM of at least three independent experiments ($n \geq 3$)

Conc.	% Inhibition			
	AA	L1	Complex 1	Complex 2
5μM	20.48	4.43	18.03	11.59
10μM	32.55	8.64	24.69	16.64
15μM	43.65	13.67	31.03	21.02
20μM	51.02	19.66	44.88	36.55
25μM	60.69	25.43	57.99	47.54
30μM	67.64	35.54	63.95	56.65
35 μM	80.59	49.66	78.14	61.44
**IC_{50} μM**	**19.95**	**36.09**	**21.73**	**28.60**

Table S9: Antibacterial activity of ligand **L1** and complexes **1** & **2** against gram positive and gram-negative bacteria.

Compounds	Max. inhibition zone, x ± SD (mm)			
	Gram–positive		Gram–negative	
	S. aureus	*B. subtilis*	*E. coli*	*P. aeruginosa*
**L1**	6 ±0.32	9 ±0.70	4± 0.43	5± 0.72
**Complex 1**	9 ±0.64	11 ±0.33	3± 0.71	7± 0.63
**Complex 2**	16 ±0.40	13 ±0.31	6± 0.64	9± 0.21

Table S10: Minimum inhibition concentration values of **L1** and complexes **1** & **2** against gram positive and gram-negative bacteria.

Compounds	Minimum inhibitory concentration (MIC) in mM			
	S. aureus	*B. subtilis*	*E. coli*	*P. aeruginosa*
**L1**	7.2	6.8	7.8	7.0
**Complex 1**	5.5	5.9	8.2	6.5
**Complex 2**	3.8	5.2	7.4	6.9

Table S11: Percent cell viability of A549 (lung cancer) cell line in a dose dependent manner when treated with **L1** and complexes **1** & **2** along with control (DMSO). A one-way analysis of variance (ANOVA) was used to calculate the data, and then the Dunnet's multiple comparison test was performed. Data represent mean ± SEM of three independent trials ($n \geq 3$); *p, **p, ***p < 0.05, 0.01, and 0.001 *vs* control group.

Sample	Conc. (µM)	1	2	3	% Cell viability
**L1**	**Control**	106.219	97.509	99.577	101.101
	**1**	107.034	107.970	114.867	109.957
	**2.5**	116.496	116.496	112.235	115.075
	**5**	116.935	112.549	113.865	114.449
	**10**	117.311	113.927	114.303	115.180
	**20**	126.084	116.371	112.674	118.376
**Complex 1**	**Control**	106.219	97.509	99.577	101.101
	**1**	119.818	112.486	117.875	116.726
	**2.5**	117.875	120.282	115.932	118.029
	**5**	112.737	116.935	103.713	111.128
	**10**	113.614	111.797	103.023	109.478
	**20**	87.169	79.711	76.390	81.090
**Complex 2**	**Control**	106.509	100.598	97.642	101.583
	**2.5**	113.687	114.637	110.520	112.948
	**5**	76.002	77.797	80.752	78.850
	**10**	59.007	65.974	80.964	51.981
	**20**	46.023	45.812	41.907	44.580

Table S12: Percent cell viability of MDA–MB–231 (triple negative breast cancer) cell line in a dose dependent manner when treated with **L1** and complexes **1** & **2** along with control (DMSO). A one–way analysis of variance (ANOVA) was used to calculate the data, and then the Dunnet's multiple comparison test was performed. Data represent mean ± SEM of three independent trials ($n \geq 3$); *p, **p, ***p < 0.05, 0.01, and 0.001 *vs* control group.

Sample	Conc. (µM)	1	2	3	% Cell viability
**L1**	**Control**	103.181	101.616	96.920	100.572
	**5**	98.621	96.647	113.731	102.999
	**10**	93.312	104.066	103.250	100.209
	**20**	102.501	101.412	101.003	101.638
**Complex 1**	**Control**	103.181	101.616	96.920	100.572
	**5**	103.522	101.480	101.684	102.228
	**10**	93.380	92.632	92.972	92.994
	**20**	85.689	80.313	75.975	80.659
**Complex 2**	**Control**	103.181	101.616	96.920	100.572
	**5**	104.611	103.590	104.407	104.202
	**10**	98.009	100.459	98.281	98.916
	**20**	72.962	80.517	73.711	75.73

Table S13: Comparison table of IC_{50} values of complex **2** with some previously reported zinc hydrazone Schiff base complexes against A549 cancer cell line.

S. No	Compound	IC_{50} (µM)	Reference
**1**	Complex **2**	17.54	Present work
**2**	$[Zn(\mathbf{L^1})Cl_2].2H_2O$	125.0	S1
**3**	$[Zn(\mathbf{L^2})Cl_2]$	103.8	S1
**4**	$[Zn(\mathbf{L^3})Cl_2]$	188.2	S1
**5**	$[Zn\mathbf{L^5}]$	18.2	S2
**6**	$[Zn\mathbf{L^4}(N_3)_2]$	~400.0	S3
**7**	Cisplatin	30 ± 5.0	S4
Where $\mathbf{L^1}$, $\mathbf{L^2}$, $\mathbf{L^3}$, $\mathbf{L^4}$ and $\mathbf{L^5}$ are hydrazone–based Schiff base ligands			

Figures

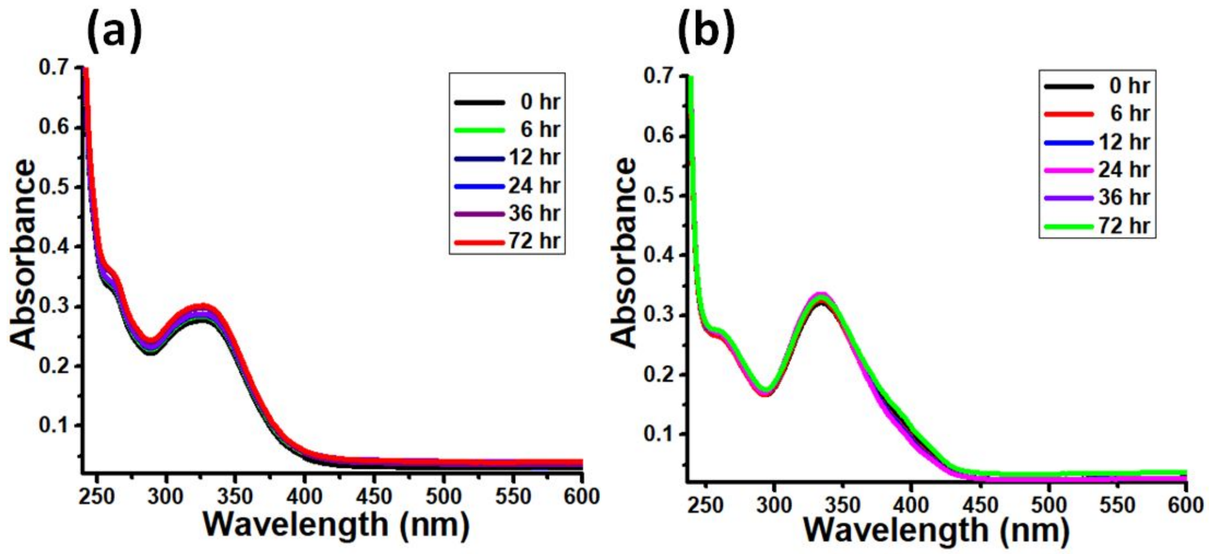

Figure S1 Solution stability studies of (a) complex **1** & (b) complex **2** at varied time intervals using UV–vis spectroscopy.

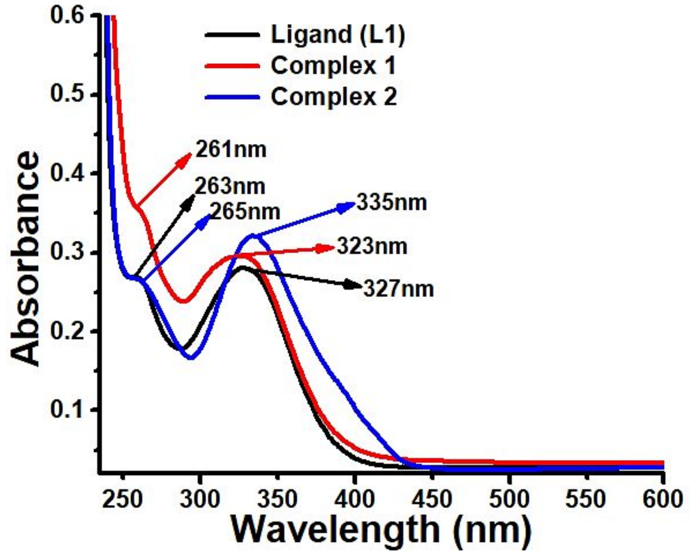

Figure S2 Comparative UV–vis spectra of ligand (**L1**) and complexes **1** & **2**.

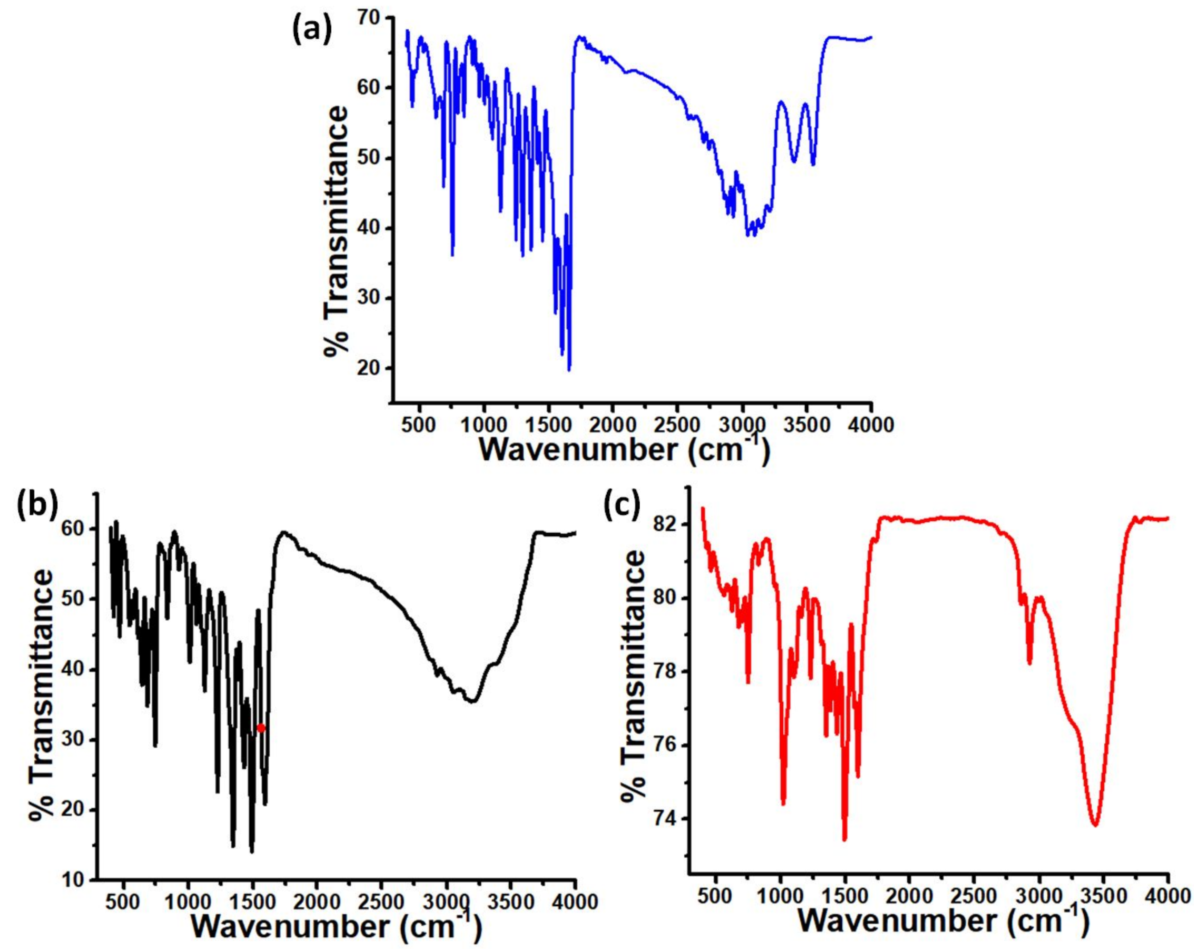

Figure S3 FTIR spectra of (a) ligand **L1,** (b) complex **1** and (c) complex **2**.

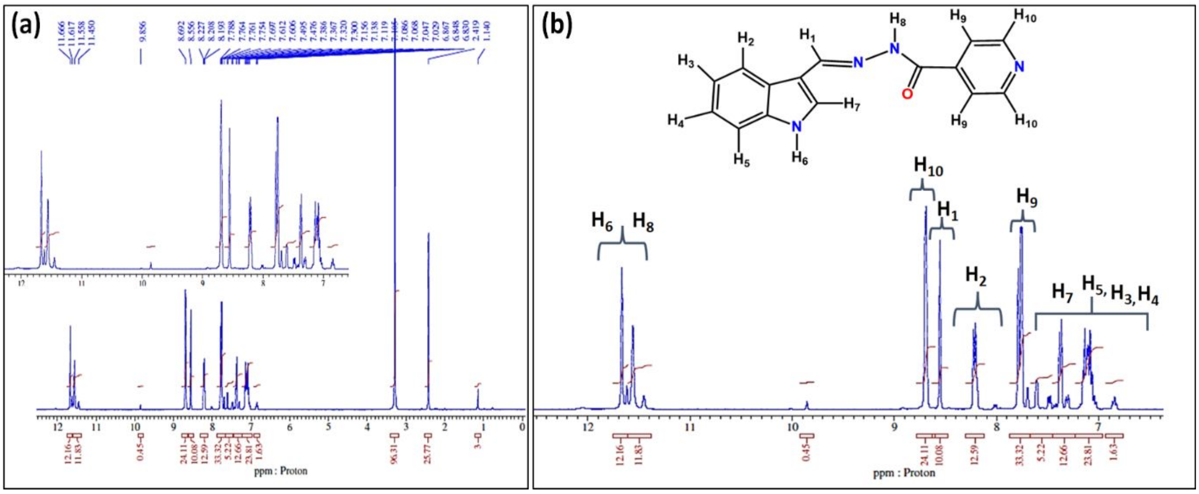

Figure S4 ^{1}HNMR spectra of ligand **L1**, (a) full view and (b) close view.

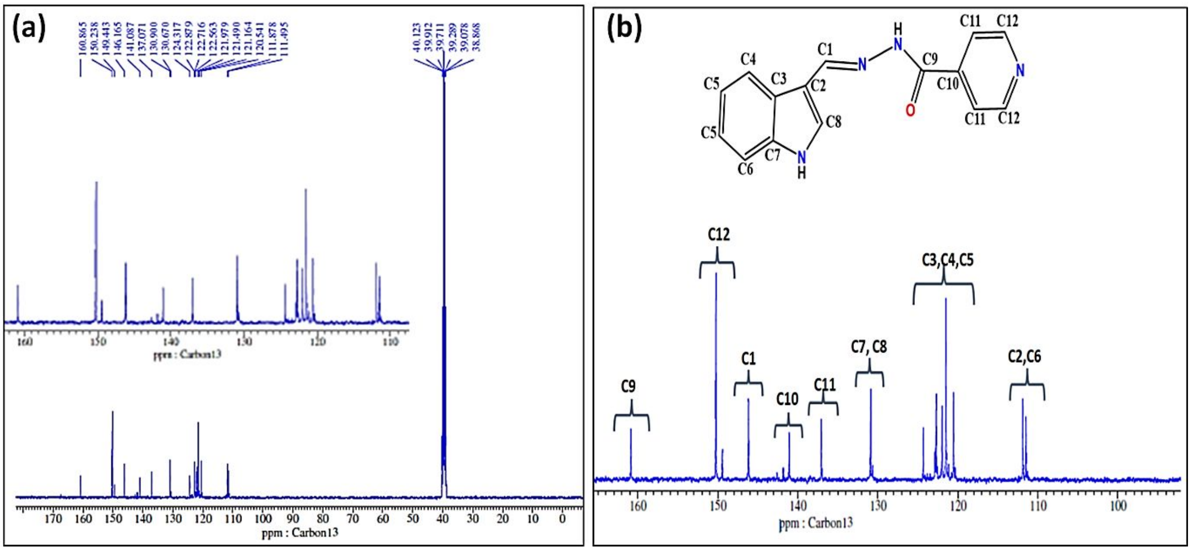

Figure S5 ^{13}CNMR of ligand **L1** (a) full view and (b) close view.

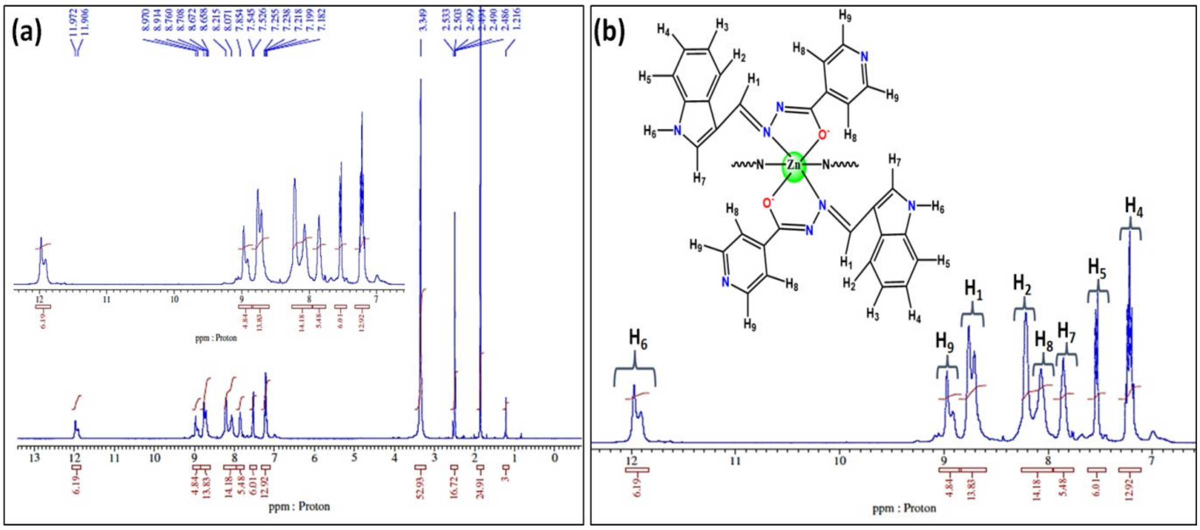

Figure S6 ^{1}HNMR of complex **2,** (a) full view and (b) close view.

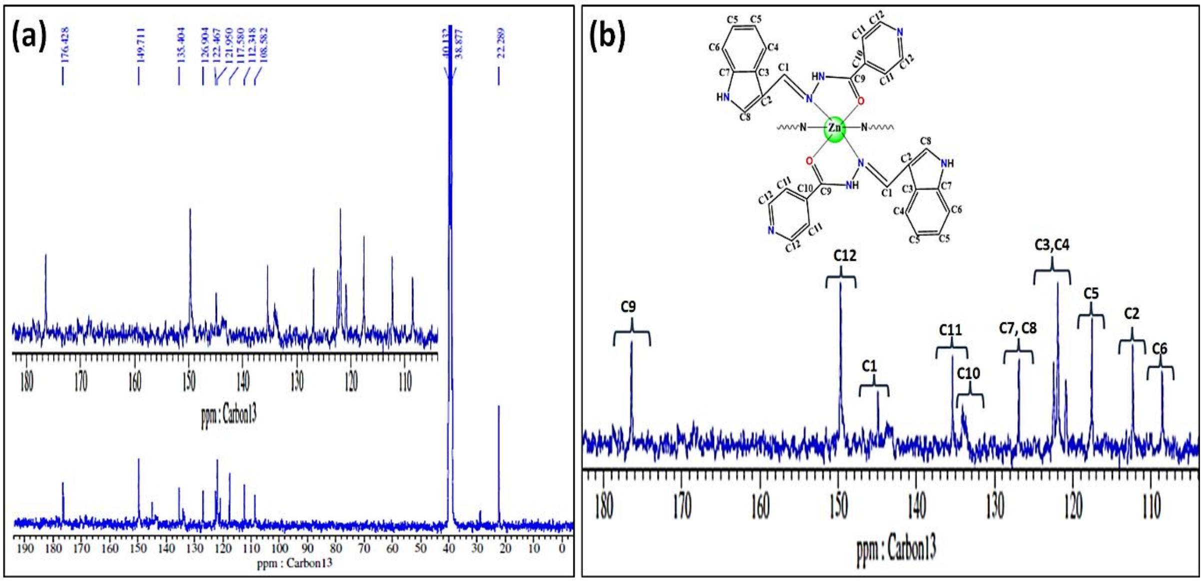

Figure S7 ^{13}CNMR of complex **2,** (a) full view and (b) close view.

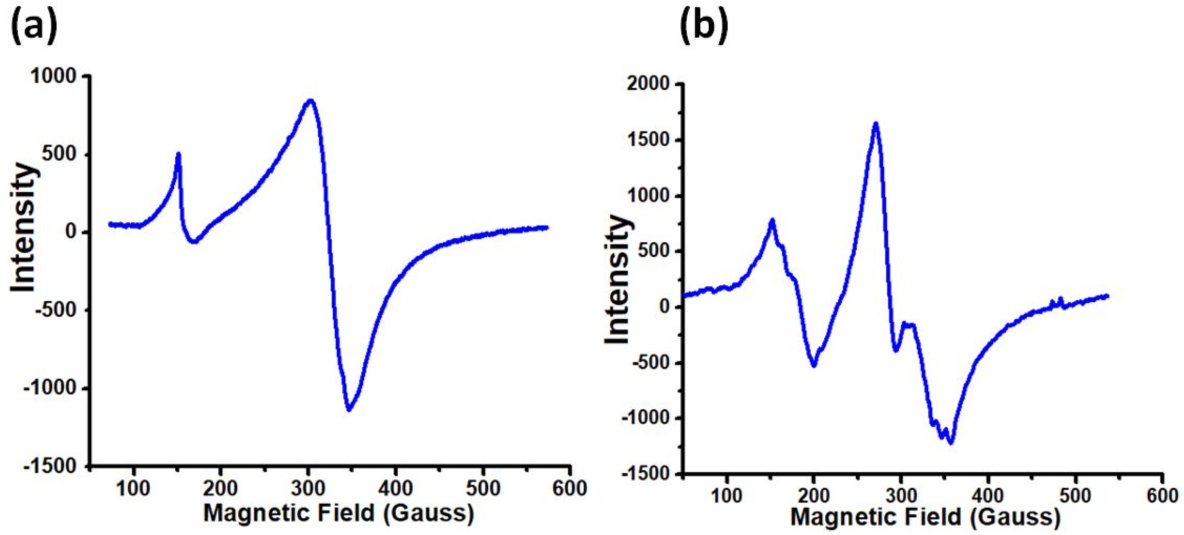

Figure S8 EPR spectra of complex **1** at (a) RT and (b) LNT.

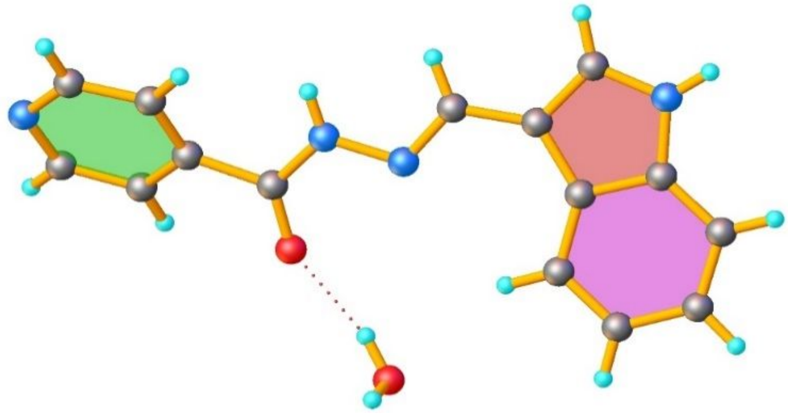

Figure S9 Single crystal-XRD structure of Schiff base ligand **L1**.

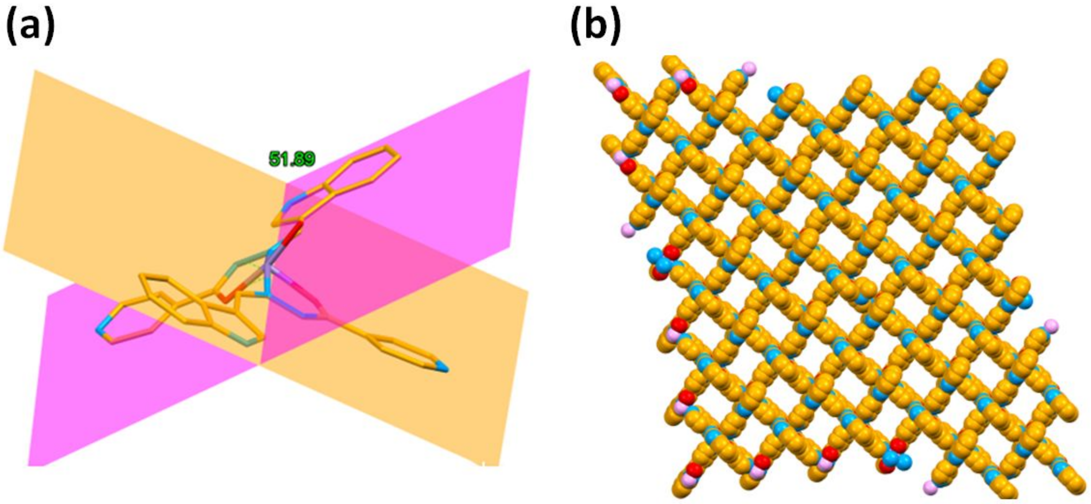

Figure S10 (a) Single XRD image illustrating dihedral angle between two planes and (b) perspective view of a space filled model of complex **2** along 2D network.

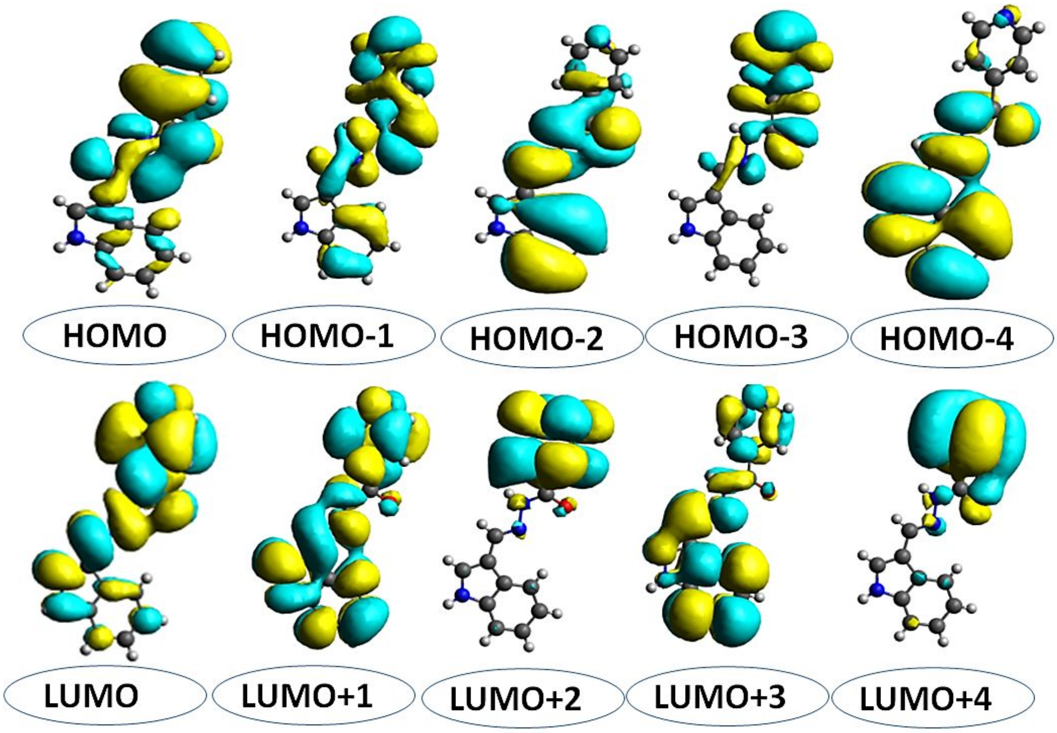

Figure S11 FMOs of ligand **L1** generated at B3LYP functional.

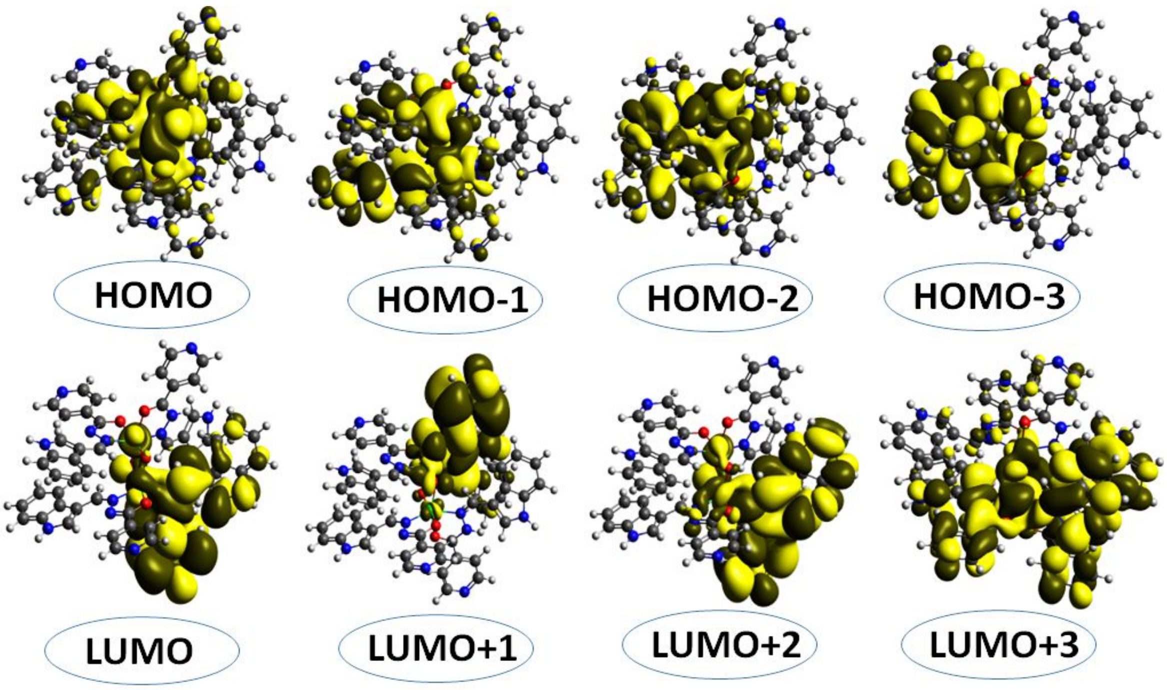

Figure S12 FMOs of complex **1** generated at B3LYP functional.

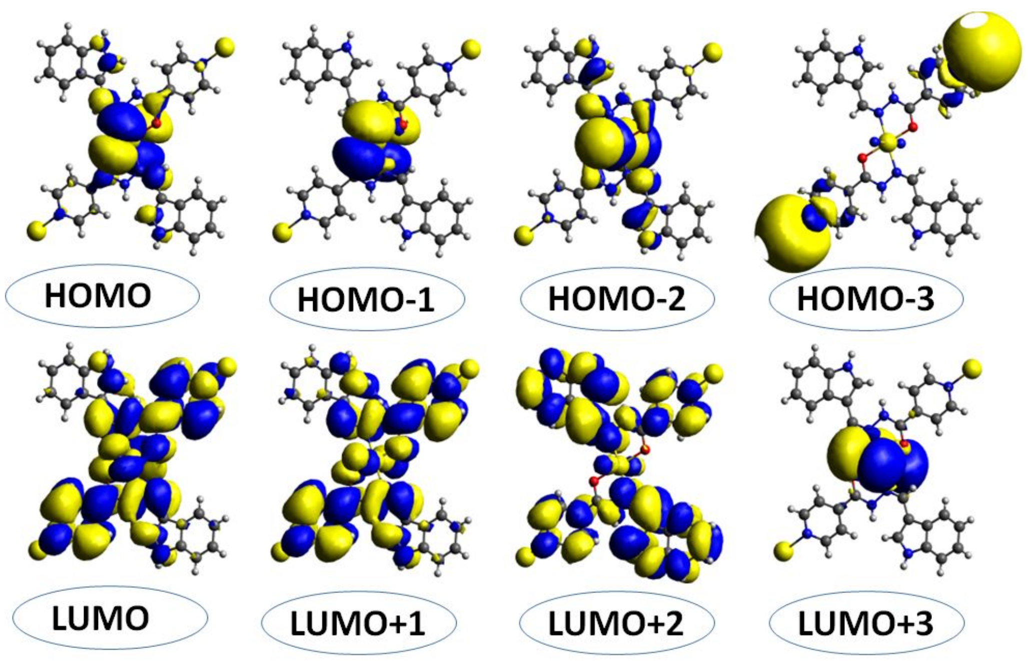

Figure S13 FMOs of complex **2** generated at B3LYP functional.

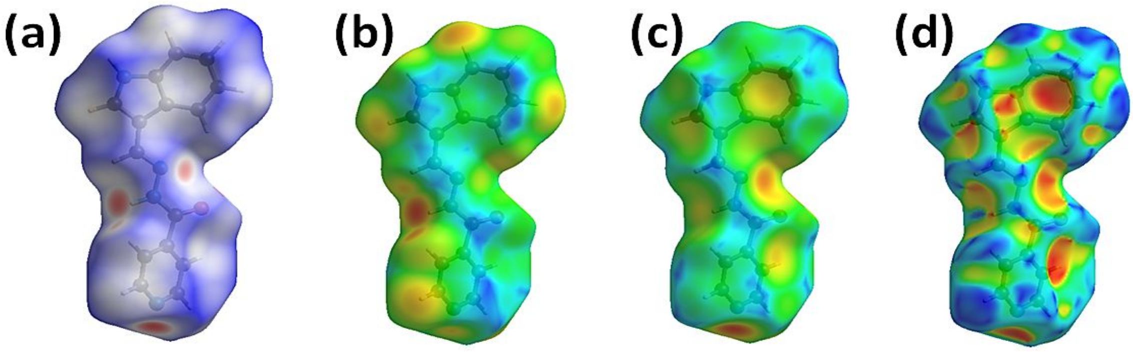

Figure S14 3D Hirshfeld surface mapping of **L1** (a) d_{norm}, (b) d_e (c) d_i and (d) shape index

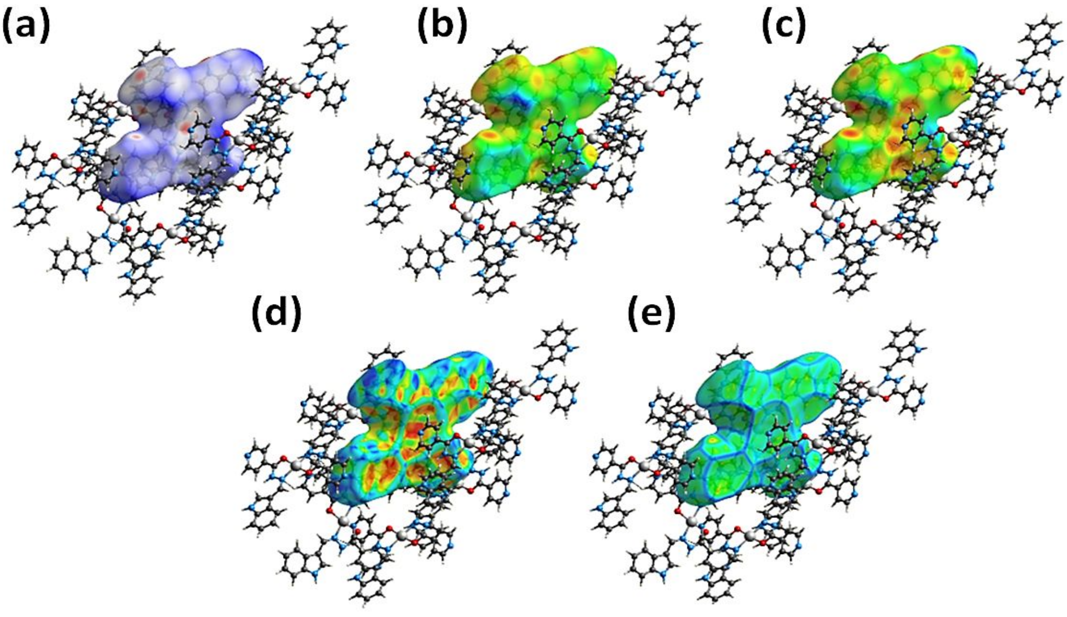

Figure S15 3D Hirshfeld surface of an asymmetric unit of complex **2** (a) d_{norm}, (b) d_e (c) d_i (d) shape index and (e) curvedness.

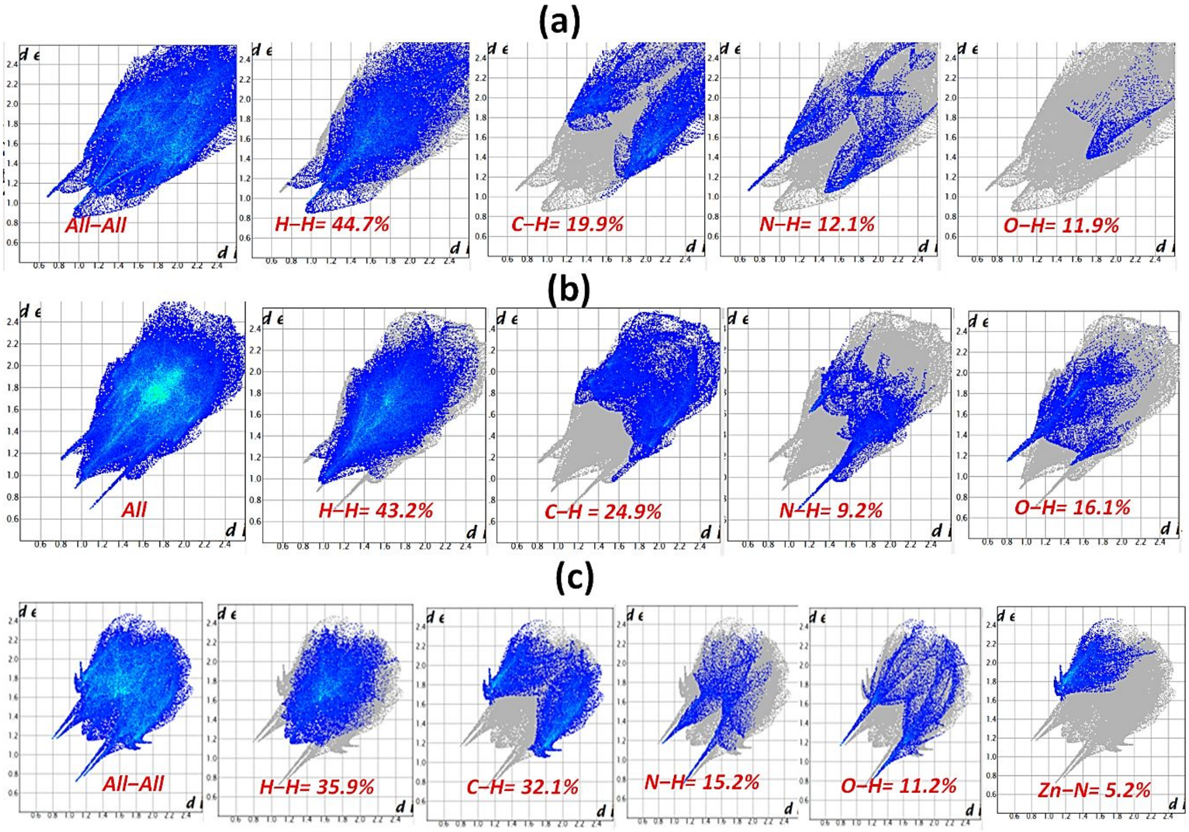

Figure S16 2D fingerprint plots of (a) **L1**, (b) complex **1** and (c) complex **2**, illustrating the major contributions of various molecular contacts to the total Hirshfeld surface.

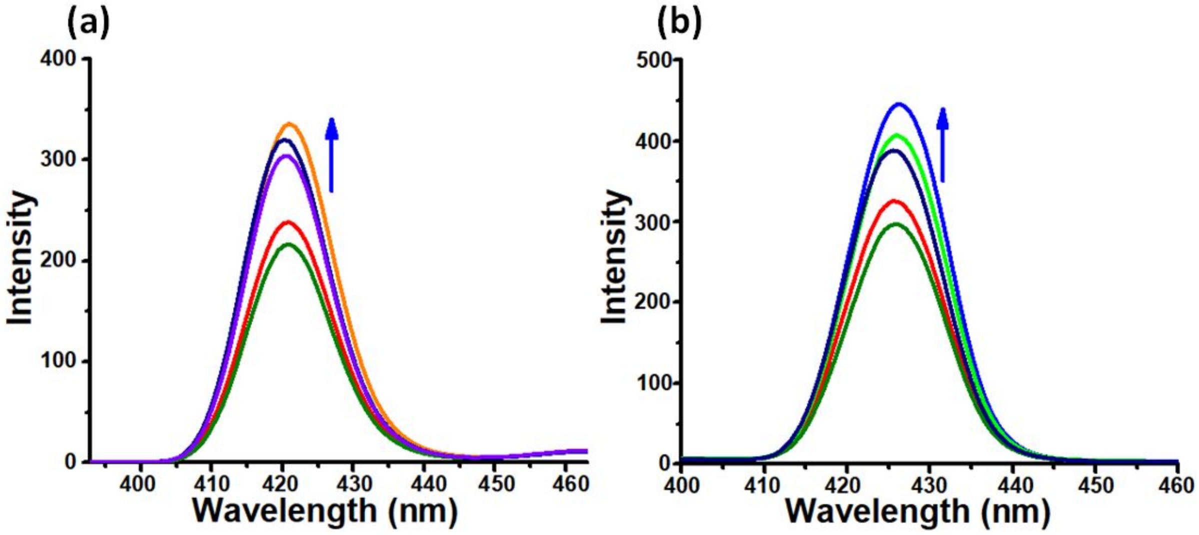

Figure S17 Emission spectra of (a) complex **1** and (b) complex **2** in presence of increasing aliquots of ct-DNA. [complex **1**] = [complex **2**] = 3 × 10^{-6} M, [DNA] = 0.1–0.5 × 10^{-5} M

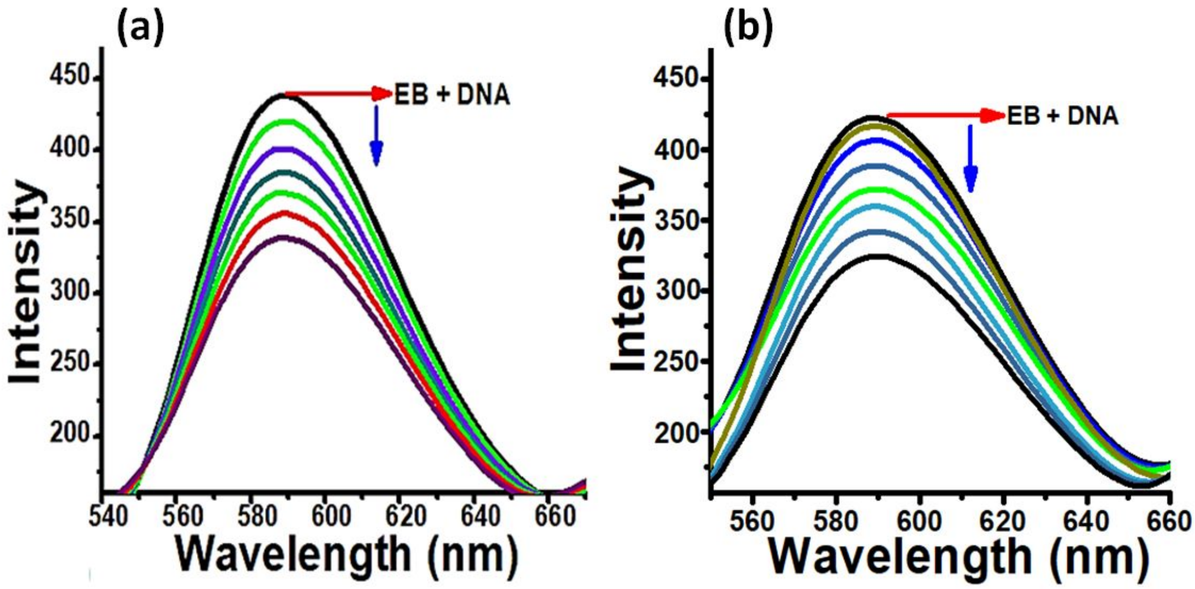

Figure S18 Emission spectra of EB–DNA system recorded in Tris-HCl buffer at pH = 7.3 (a) in presence of complex **1** (b) in presence of complex **2**. [EB] = [DNA] = 2 × 10^{-6} M.

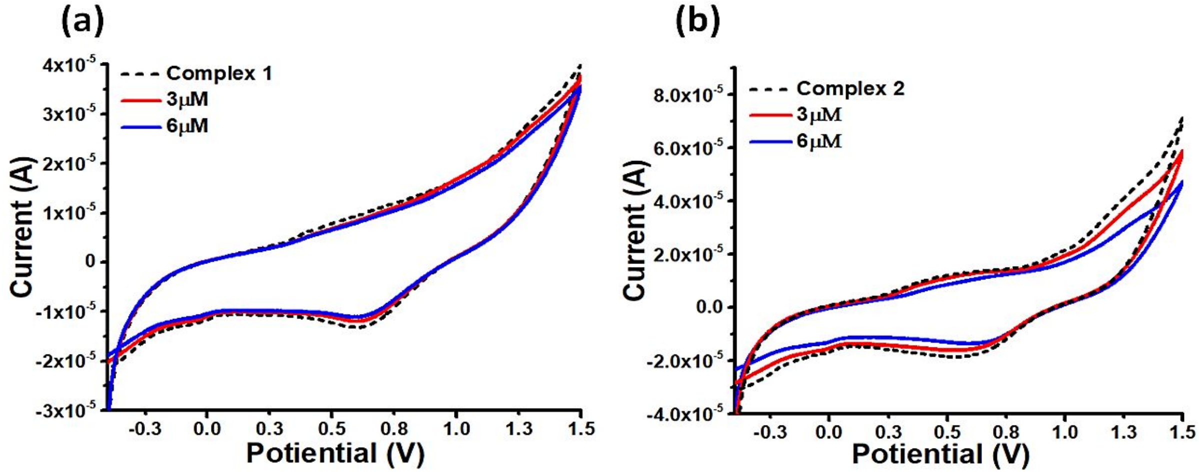

Figure S19 Cyclic voltammogram curves of complexes (a) complex **1** and (b) complex **2** in presence of ct-DNA in Tris HCl buffer of pH=7.3. [Complex **1**] = [Complex **2**] = 3 × 10^{-6} M, [DNA] 0.3–0.6 × 10^{-5} M. Scan rate = 100 mVs^{-1}.

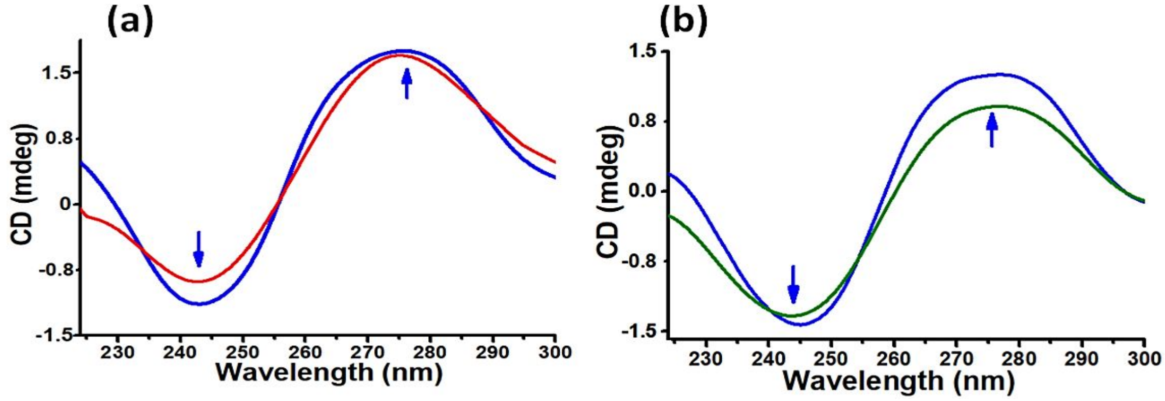

Figure S20 CD spectra of ct-DNA (red), (a) ct-DNA + complex **1** (blue) and (b) ct-DNA + complex **2** (blue) in Tris–HCl buffer (pH=7.3). [DNA] = 0.4 × 10^{-5} M and [complex **1**] = [complex **2**] = 2 × 10^{-5} M.

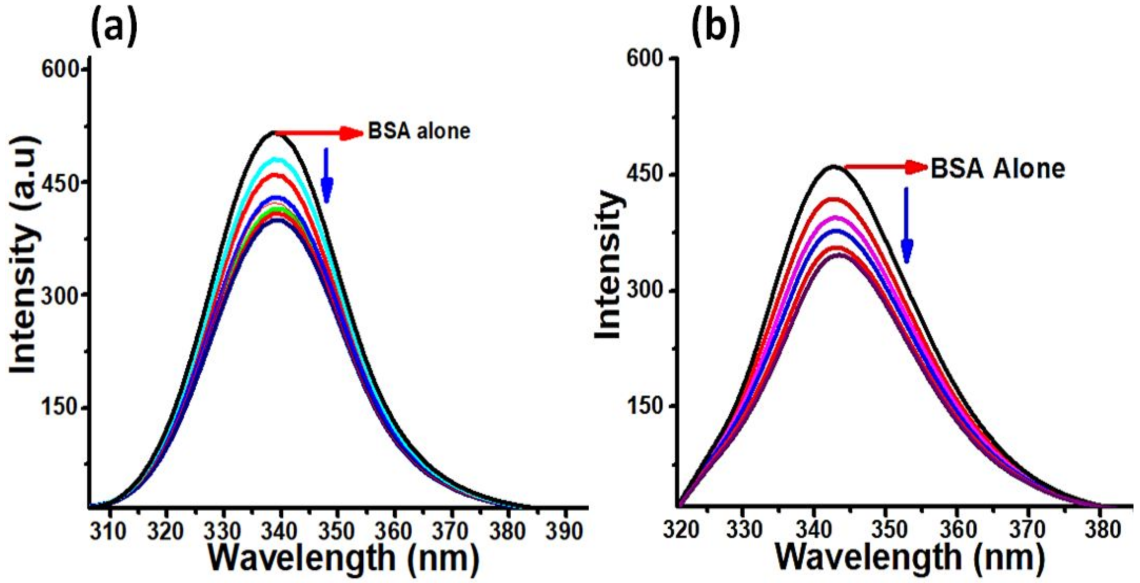

Figure S21 Emission spectra of BSA in the presence and absence of (a) complex **1** and (b) complex **2**. Arrows indicate quenching of emission maxima in presence of complexes **1** & **2**. [BSA]= 3 × 10^{-5}M, [complex **1**] = [complex **2**] = 0.1–0.6 × 10^{-5}M.

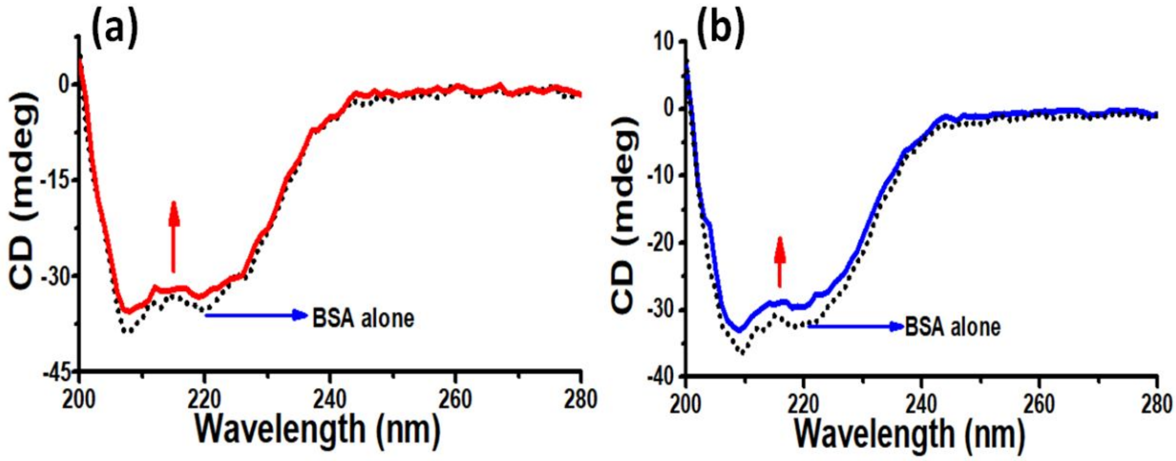

Figure S22 CD spectrum of BSA alone (dotted line) in the presence and absence of (a) complex **1** (red) and (b) complex **2** (blue). [BSA] = 3 × 10^{-6} M and [complex] = 0. 2 × 10^{-5} M.

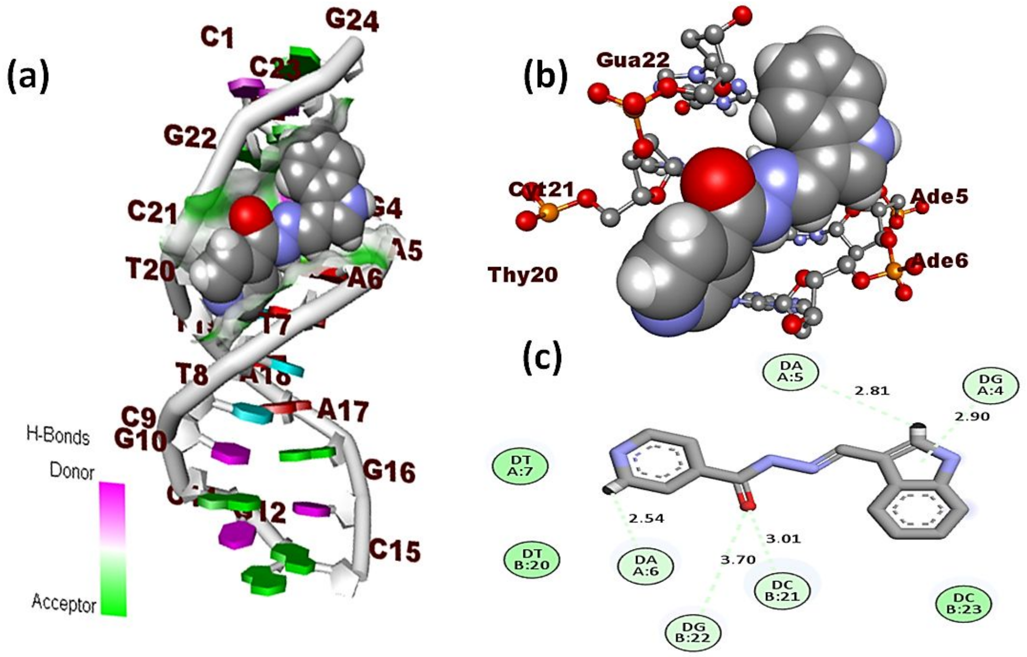

Figure S23 Molecular docked structure of **L1** (a) fitted inside the DNA dodecamer duplex of sequence d(CGCGAATTCGCG)$_2$ (PDB ID: 1BNA); (b) ligand nucleotide interaction in 3D view (c) interaction with different nucleotides in 2D view.

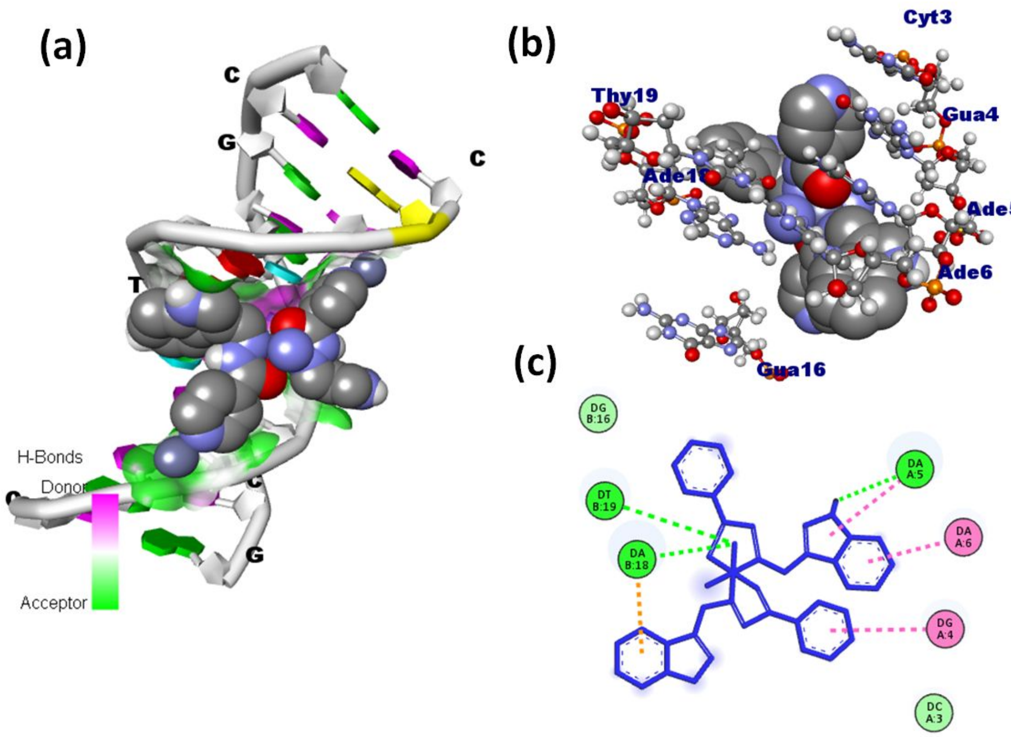

Figure S24 Molecular docked structure of complex **2** (a) fitted inside the DNA dodecamer duplex of sequence d(CGCGAATTCGCG)2 (PDB ID: 1BNA); (b) ligand nucleotide interaction (c) interaction with different nucleotides in 2D view.

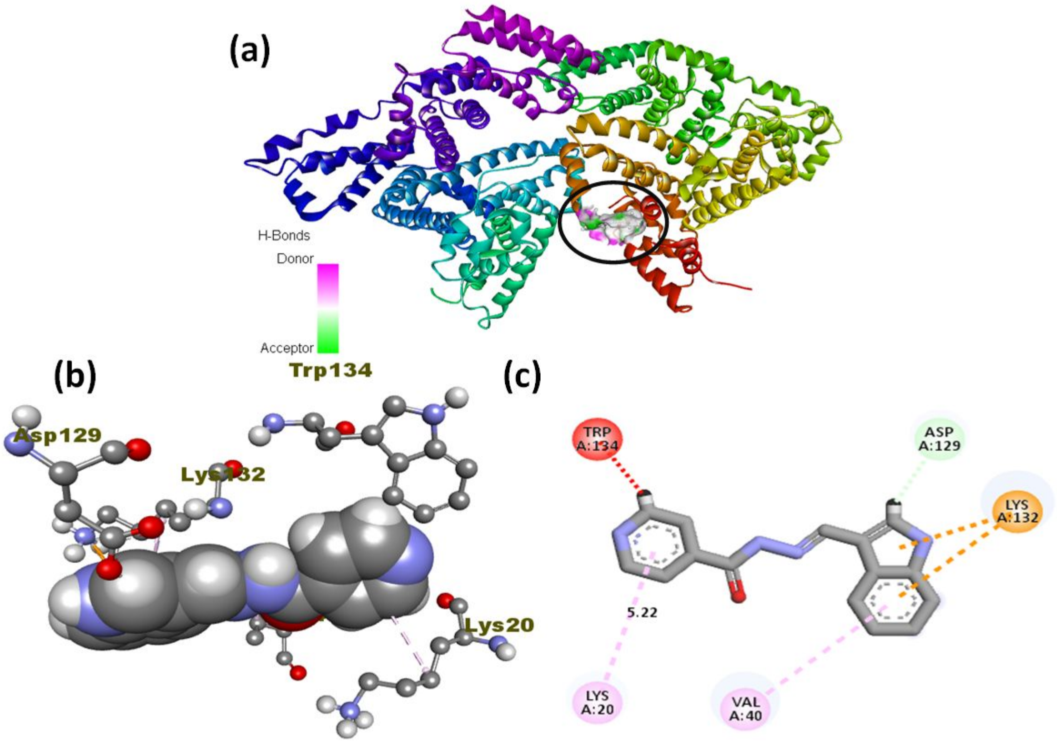

Figure S25 (a) Molecular docked model poses of **L1** located within the hydrophobic pocket in subdomain IIA of BSA stabilized by intricate hydrogen bonding interactions and van der walls forces, (b) interaction of **L1** with amino acid residues showed in 3D view and (c) interaction of **L1**with amino acid residues showed in 2D view.

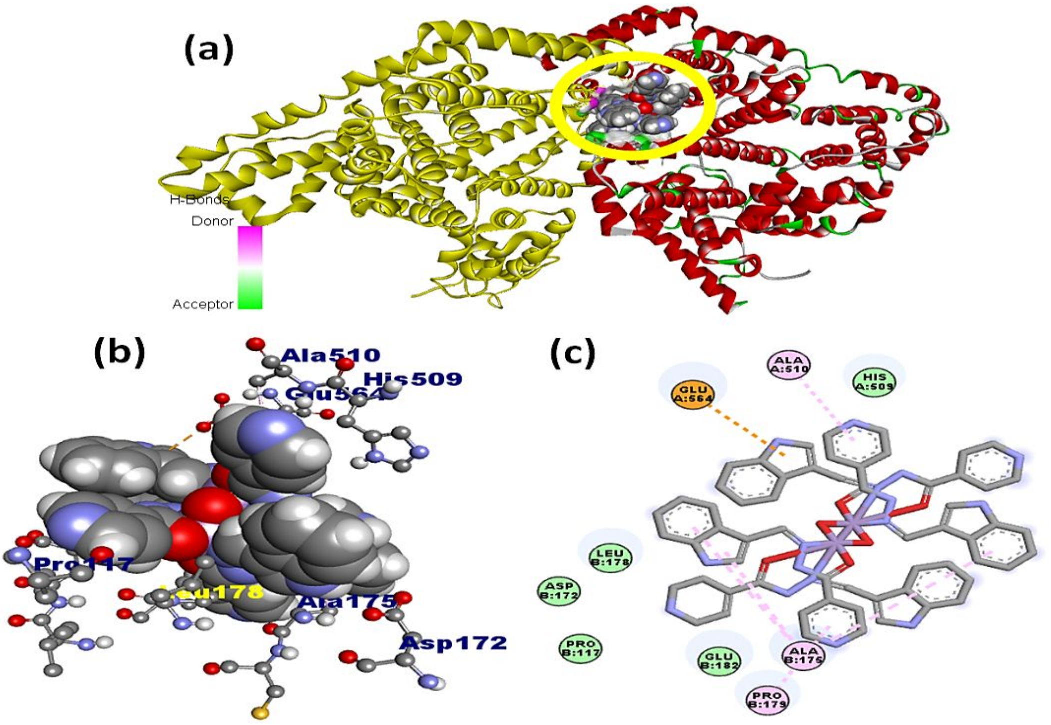

Figure S26 (a) Molecular docked model of complex **1** located within the hydrophobic pocket in subdomain IIA of BSA stabilized by intricate hydrogen bonding interactions and van der walls forces, (b) interaction of complex **1** with amino acid residues showed in 3D view and (c) interaction of complex **1** with amino acid residues showed in 2D view.

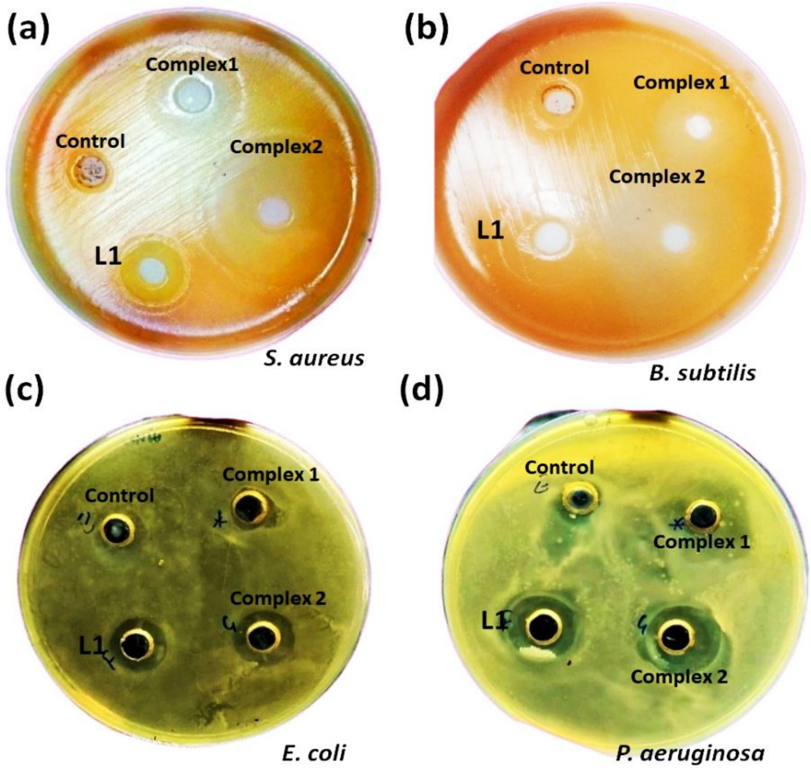

Figure S27. Comparative antibacterial action of control (solvent), ligand (**L1**) and complexes **1** & **2** against (a & b) gram positive (*S. aureus* and *B. subtilis*) and (c & d) gram-negative (*E. coli* and *P. aeruginosa*) bacterial strains.

References

(S1) Dasgupta, S.; Karim, S.; Banerjee, S.; Saha, M.; Saha, K. D.; Das, D. Designing of novel zinc (II) Schiff base complexes having acyl hydrazone linkage: study of phosphatase and anti-cancer activities. *Dalton Trans.* **2020**, 49, 1232−1240.

(S2) Chang, H. Q.; Jia, L.; Xu, J.; Xu, Z. Q.; Chen, R. H.; Wu, W. N.; Bie, H. Y.; Zhu, T. F.; Ma, T. L.; Wang, Y. Syntheses, characterizations, antitumor activities and cell apoptosis induction of Cu (II), Zn (II) and Cd (II) complexes with hydrazone Schiff base derived from isonicotinohydrazide. *Inorg. Chem. Commun.* **2015**, 57, 8−10.

(S3) Stevanovic, N.; Zlatar, M.; Novaković, I.; Pevec, A.; Radanović, D.; Matić, I. Z.; Crnogorac, M. D.; Stanojković, T.; Vujcic, M.; Gruden, M.; Sladic, D.; Anđelkovic, Iztok T. K.; Cobeljic, B. (). Cu (II), Mn (II) and Zn (II) complexes of hydrazones with a quaternary ammonium moiety: synthesis, experimental and theoretical characterization and cytotoxic activity. *Dalton Trans.* **2022**, 51, 185−196.

(S4) Baharuddin, P.; Satar, N.; Fakiruddin, K. S.; Zakaria, N.; Lim, M. N.; Yusoff, N. M.; Zakaria, Z.; Yahaya, B. H. Curcumin improves the efficacy of cisplatin by targeting cancer stem-like cells through p21 and cyclin D1-mediated tumour cell inhibition in non-small cell lung cancer cell lines. *Oncol. Rep.* **2016**, 35, 13–25.
